# Temporally sequenced anticancer drugs overcome adaptive resistance by targeting a vulnerable chemotherapy-induced phenotypic transition

**DOI:** 10.1038/ncomms7139

**Published:** 2015-02-11

**Authors:** Aaron Goldman, Biswanath Majumder, Andrew Dhawan, Sudharshan Ravi, David Goldman, Mohammad Kohandel, Pradip K. Majumder, Shiladitya Sengupta

**Affiliations:** 1 Department of Medicine, Harvard Medical School, Boston, Massachusetts 02115, USA; 2 Harvard-MIT Division of Health Sciences and Technology, Cambridge, Massachusetts 02139, USA; 3 Division of Biomedical Engineering, Department of Medicine, Brigham and Women’s Hospital, Boston, Massachusetts 02115, USA; 4 India Innovation Research Center, Bangalore 560099, India; 5 Mitra Biotech Pvt Ltd, Narayana Nethrayala, Bangalore 560099, India; 6 School of Medicine, Queen’s University, Kingston, Ontario, Canada K7L 3N6; 7 7730E BlackCrest Pl., Tucson, Arizona 85750, USA; 8 Department of Applied Mathematics, University of Waterloo, Waterloo, Ontario, Canada N2L 3G1; 9 Dana Farber Cancer Institute, Boston, Massachusetts 02115, USA

## Abstract

Understanding the emerging models of adaptive resistance is key to overcoming cancer chemotherapy failure. Using human breast cancer explants, *in vitro* cell lines, mouse *in vivo* studies and mathematical modelling, here we show that exposure to a taxane induces phenotypic cell state transition towards a favoured transient CD44^Hi^CD24^Hi^ chemotherapy-tolerant state. This state is associated with a clustering of CD44 and CD24 in membrane lipid rafts, leading to the activation of Src Family Kinase (SFK)/hemopoietic cell kinase (Hck) and suppression of apoptosis. The use of pharmacological inhibitors of SFK/Hck in combination with taxanes in a temporally constrained manner, where the kinase inhibitor is administered post taxane treatment, but not when co-administered, markedly sensitizes the chemotolerant cells to the chemotherapy. This approach of harnessing chemotherapy-induced phenotypic cell state transition for improving antitumour outcome could emerge as a translational strategy for the management of cancer.

## Abstract (web summary)

Adaptive resistance is an emerging cause of chemotherapy failure in cancer. Here the authors show that adaptive resistance to taxanes is mediated by the upregulation of SFK/Hck survival signalling, and that sequential administration of taxanes and SFK/Hck inhibition restores tumor cell chemosensitivity.

## Graphical abstract

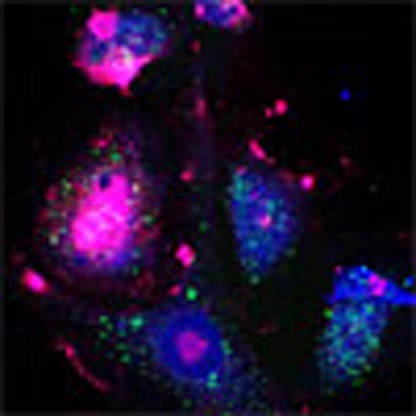

Resistance to chemotherapy is the major cause of relapse and mortality due to cancer. Darwinian principles of fitness-selected genetic mutations underscored the archetypal paradigm for acquired resistance to chemotherapy^1^. For example, mutations leading to structural changes in drug target proteins, upregulation of drug-efflux proteins or the activation of alternate survival pathways can all lead to chemotherapy failure^2^. However, recent evidences have implicated both intrinsic and adaptive resistance governed by epigenetic alterations of cancer cells in non-Darwinian relapse^3^. For example, cancer cells in patients treated with either cytotoxic or targeted agents, such as a taxane or imatinib, can exhibit drug resistance, and even grow during treatment, despite the absence of resistance-conferring genetic alterations^4^,^5^. In addition, clinical evidence exists to show that cancer cells can become resensitized to chemotherapy after a ‘drug holiday’^6^. Indeed, similar transient adaptive resistance to antibiotics has been reported in bacteria, leading to the generation of ‘persisters’^7^. Improved understanding of intrinsic and adaptive resistance is therefore the key to a successful chemotherapeutic outcome.

Early explanations of intrinsic resistance emphasized a phenotypically distinct subset of cancer stem-like cells (CSC)^8^. However, there is an increasing realization that a higher degree of intratumoral heterogeneity exists beyond CSCs, as an outcome of stochastic gene expression^9^ or due to non-genetic cell state dynamics arising from spontaneous switching between cell states within a clonal population^10^. Recent studies have revealed that phenotypic state transitions could be a consequence of external cues, including radiation and chemotherapy^3^. These findings support the hypothesis that cancer cells could potentially, phenotypically transition to a chemotolerant state, which can offer an initial survival advantage against chemotherapy in the absence of Darwinian resistance-conferring mutations. Therapeutic regimens that perturb such cell state transitions could evolve as important and clinically applicable strategies to overcome resistance. We tested this hypothesis in the context of the development of adaptive resistance to docetaxel (DTX) in breast cancer, which remains the second most common cause of cancer deaths in women^11^, and is treated with taxane-based chemotherapy^12^.

We report here that treatment of cancer cells with high concentration of taxanes results in the generation of ‘persister’ cells that are defined by a transition towards a CD44^Hi^CD24^Hi^ expression status. Using mathematical modelling and further experimental validation, we demonstrate that these cells arise as a result of chemotherapy-induced phenotypic transitions from a non-CSC population, and can confer drug resistance. This phenotypic shift correlates with the activation of the Src family kinase (SFK)/Hck pathway, and post-treatment with a SFK/Hck inhibitor within a defined temporal window enhances cell death. The concept of therapy outcome being dependent on the sequence of administration of chemotherapy agents is an emerging paradigm^13^,^14^. Our results indicate that a drug pair administered in the right temporal sequence combinations, where the leading drug induces a phenotypic cell state transition thereby uncapping a vulnerability tractable by the partner agent, could overcome adaptive resistance and enhance cell death.

## Results

### Drug-induced phenotypic transition in explants

**Figure 1:**
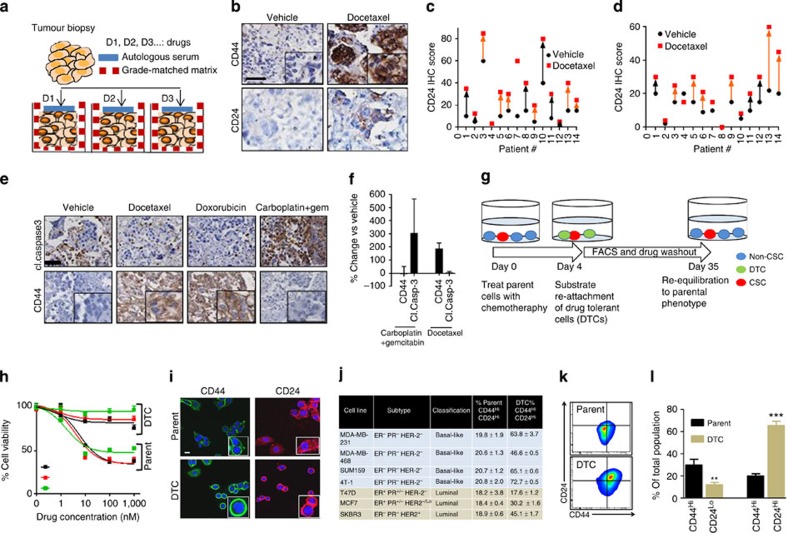
Taxane chemotherapy induces phenotypic cell state transition and adaptive resistance. (**a**) Schematic of human explant model to evaluate response of refractory human tissue to anticancer agents. Tumour biopsies were cut into ~200 mM-thick sections and cultured in microwells coated with tumour matrix and media supplemented with autologous serum. (**b**) Representative immunohistochemistry (IHC) of primary human breast tumour explants shows induction of CD44 and CD24 cell surface expression following 72 h treatment with docetaxel versus vehicle. × 40 Scale bar, 50 μm inset show higher magnification, × 100 (**c**,**d**) Graph shows quantification of CD44 and CD24 levels in the primary tumour explant studies, (*N*=14 patients). Black and red points denote the protein levels measured by IHC score in a tumour explant in vehicle- and docetaxel-treated groups. Each number denotes a patient. The orange arrows denote patients who were taxane-treatment naive, whereas those denoted with black arrows received a taxane. (**e**) Representative IHC from explant culture shows effect of different drug treatments (3.4 μM docetaxel, 5.6 μM doxorubicin) on the expression of CD44 and cleaved (cl) caspase 3 in corresponding serial sections. Gem, gemcitabine. × 40 magnification Scale bar, 50 μm. Inset shows higher magnification × 100 (**f**) Graph shows the quantification of CD44 and cleaved caspase 3 expression in the explants treated with docetaxel (*n*=9) or a combination of gemcitabine+carboplatin (*n*=2). Data shows mean±s.e.m. (**g**) Schematic shows generation of drug-tolerant cells (DTCs) selected acutely using high-concentration docetaxel chemotherapy *in vitro.* Cells were cultured in 100 μM (~20X IC_50_) docetaxel. Cells surviving by day 4 were quiescent and considered as drug-tolerant cells (DTCs). Growing out the DTCs over 35 days resulted in restoring parental properties. (**h**) Graph shows MTS cell viability analysis of parental cells and DTCs generated from of MDA-MB-231 breast cancer cells following incubation (48 h) with different tubulin-binding chemotherapeutics at indicated concentration range. (**i**) Confocal images show expression levels of CD44 and CD24 in parental cells and DTCs generated from MDA-MB-468s. Scale bar, 18 μm (**j**) The population percentage of CD44^Hi^CD24^Hi^ cells in parental and DTCs generated from an array of luminal and basal breast cancer cell lines. Data shown are mean±s.e.m., *n*=3 (*P*<0.01 other than T47D cells). (**k**) Representative FACS plot of CD44 and CD24 in MDA-MB-231 parent cells and DTC. (**l**) Graph shows quantification of CD44^Hi^/CD24^Lo^ and CD44^Hi^/CD24^Hi^ as % of total population of MDA-MB-231 parent cells and DTCs (Data shown are mean±s.e.m., *n*=8, ANOVA analysis *P<0.01, ***P<0.001).

To elucidate the mechanisms underlying adaptive resistance to anticancer therapy, we used three-dimensional explants derived from fresh tumour biopsies from patients. Three-dimensional tumour explants are emerging as powerful models to study tumour biology, as they preserve the tumour heterogeneity and microenvironment^15^. In a recent study, we have observed that culturing the explants in autologous serum and in grade-matched tumour matrix conserves the parental tumour genotypic and phenotypic characteristics^16^. We included breast cancers of different stages and receptor status, including those that were taxanes-treatment naive (Supplementary Table 1). We used 200 μm tumour explants in this study as drugs can diffuse through such thickness^17^ (Fig. 1a). CD44, a membrane glycoprotein, has been associated with chemorefractory, more mesenchymal stem-like characteristics^8^,^18^. In contrast, CD24-positive breast cancer cells have been reported to be more of the differentiated luminal and a Her2+ subtype, whereas basal-like tumours were classified as CD24^−/Lo^ (ref. 19). We observed a significant inter-tumoral heterogeneity in the baseline expression of CD44 and CD24, and the distribution was normal between tumours from taxane-treated and taxane-naive patients (Fig. 1b–d). Interestingly, incubating the explants with high-concentration DTX (3.4 μM)^20^ for 72 h resulted in an increase in the median expression of both CD44 and CD24 as compared with vehicle-treated explants (*P*<0.01) (Fig. 1b–d), irrespective of the tumour type. In addition, the DTX-induced increase in expression of CD24 and CD44 was similar in explants generated from tumours that had progressed clinically on taxanes and those generated from taxanes-treatment naive patients, indicating that the phenotypic plasticity did not rely on the acquisition of resistance. The upregulation of CD44 following DTX treatment was correlated with reduced apoptosis as seen in decreased cleaved caspase-3 levels compared with baseline (Fig. 1e,f). Treatment with doxorubicin, which is widely used in the adjuvant or metastatic settings in breast cancer, similarly induced CD44 expression with reduced cleaved caspase-3 levels. Interestingly, in contrast, treatment with carboplatin (100 μM) and gemcitabine (100 μM) induced apoptosis without any upregulation of CD44 expression (Fig. 1e,f). Indeed, in a recent study, a combination of gemcitabine and carboplatin was found to be effective for pretreated patients with metastatic breast cancer^21^.

### Drug-induced phenotypic plasticity and tolerance

The results from explant studies suggested that taxane and anthracycline chemotherapy induce a phenotypic transition to a chemotherapy-refractory CD44^Hi^CD24^Hi^ state, rather than selecting for ‘privileged’ subsets. We recapitulated these findings using an array of established luminal and basal-like breast cancer cell lines. Although the IC_50_ values of DTX in cancer cells typically range between high pM and low nM range^20^, a subset of the treatment-naive parent cell population was found to survive at supramaximal concentrations ≥100 nM of DTX. These persister cells were termed as drug-tolerant cells (DTCs) (Fig. 1g,h), and were characterized by low baseline apoptosis (Supplementary Fig. 1a,b). The DTCs showed cross-tolerance to other cytotoxics, including doxorubicin, vincristine and cabazitaxel (Fig. 1h). Cabazitaxel, a recently approved taxane for treatment of hormone-resistant prostate cancer, has poor affinity for drug-efflux *p*-glycoproteins, suggesting that the resistance of DTCs to the cytotoxics is independent of drug-efflux^22^. This was further validated by treating the cells with elacridar, a *p*-glycoproteins-transport inhibitor, which failed to reverse the resistance to the cytotoxics (Supplementary Fig. 1c,d). Furthermore, no changes in MDR1 expression were noted between parent cells and DTCs (Supplementary Fig. 1e).

We next explored whether the DTCs exhibit higher levels of CD44 and CD24. As shown in Fig. 1i, confocal imaging revealed an enhanced membrane expression of CD44 and CD24 in the DTCs derived from the basal-like breast cancer cell line MDA-MB-468 as compared with treatment-naive parent cells, which was validated using fluorescence-activated cell sorting (FACS) (Fig. 1j). In the context of breast cancer, a CD44^Hi^CD24^−/Lo^ cell has classically been defined as a belonging to the cancer stem cell population that confers intrinsic resistance^8^. Interestingly, consistent with the observation in the MDA-MB-468s, we observed an increase in the CD44^Hi^CD24^Hi^ population in the DTCs that were derived from MDA-MB-231 (basal), SUM159 (basal) or 4T1 (basal, murine) cells. A similar increase in the CD44^Hi^CD24^Hi^ population was also observed in the DTCs generated from the luminal cell lines, MCF7 and SKBr3, but not in the T47D cell line (Fig. 1j–l). Additional studies revealed an increase in CD44 expression in the DTCs generated from melanoma cell line MDA-MB-435 and murine ovarian cell lines, 4306 and 4412 (Supplementary Fig. 2a), suggesting that this phenomenon is not restricted only to breast cancer. CD44 was also found to be elevated following treatment with doxorubicin (Supplementary Fig. 2b). We did not observe an increase in the percentage of CD44^Hi^CD24^Lo^ population in the DTCs compared with the parent cells (Fig. 1l).

As the high concentration of DTX also induced cell death in parent cells, it was not evident whether the increase in the CD44^Hi^CD24^Hi^ phenotype was a true induction or just an enrichment of the subtype as in the case of CSCs. To dissect this, we treated parent cells acutely (24 h) with low-dose chemotherapy (10 nM for MDA-MB-468 and 25 nM for MDA-MB-231, respectively) that had no effect on cell viability, that is, did not select for ‘privileged’ cells (Supplementary Fig. 3a,c). Interestingly, as shown in Supplementary Fig. 3b,d, this resulted in an increase in the CD44^Hi^ and CD24^Hi^ populations. Furthermore, we observed a dose-dependent induction towards the CD44^Hi^CD24^Hi^ phenotype (Supplementary Fig. 3e,f). Taken together, these results suggest that the observed chemotherapy tolerance could potentially arise from drug-induced phenotypic cell state transition, distinct from the established models of clonal selections of privileged subsets.

### Quantitative model of phenotypic cell state transitions

**Figure 2:**
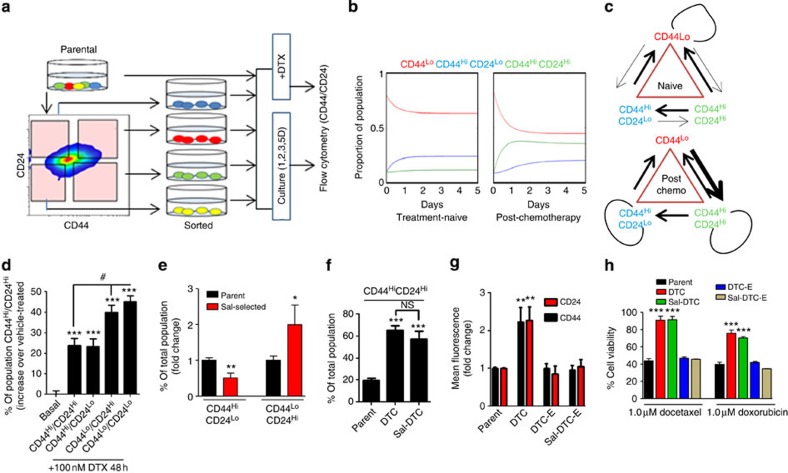
Modelling the induction of CD44^Hi^CD24^Hi^ cells. (**a**) Schematic shows experimental design used to derive mathematical parameters of population dynamics. Treatment of MDA-MB-231 breast cancer cells with 25 nM docetaxel (DTX) for 24 h induces phenotype plasticity rather than providing a selection pressure. In parallel, starting cells with different permutations and combinations of CD24 and CD44 expression levels were used, and the expression of CD44CD24 was monitored over defined time points. (**b**) Population dynamics modelling derived from experimental data indicates temporal kinetics of breast cancer cells in distinct compartments over 5 days (CD44^Lo^ described as non-CSC). Left panel shows dynamics of distinct phenotypes under basal conditions, right panel demonstrates population dynamics under chemotherapy pressure. (**c**) Schematic shows subpopulation transition dynamics and predictive contribution of each population under chemotherapy pressure or basal state, saturated to equilibrium. Arrow weights denote prevalence of conversion. Loops indicate propensity to replicate or transition. (**d**) Treatment-naive 231-parental cells were sorted into CD44^Hi^CD24^Hi^, CD44^Hi^CD24^Lo^, CD44^Lo^CD24^Hi^ and CD44^Lo^CD24^Lo^ subpopulations, which were subsequently exposed to high-dose docetaxel (100 nM) for 48 h and re-analyzed by FACS for CD44^Hi^CD24^Hi^ subset expressed as % of total population. ‘Basal’ denotes the change in % of CD44^Hi^CD24^Hi^ subset in parental cells treated with vehicle. Data are mean±s.e.m. (ANOVA analysis, *N*=7, #*P*<0.05, *P<0.05 **P<0.01 versus basal group). (**e**) Depletion of intrinsic CSC population with salinomycin (5 μM, 48 h) was confirmed by reduction of a CSC (CD44^Hi^/CD24^Lo^) and enrichment of a non-CSC phenotype (CD44^Lo^CD24^Hi^) expressed as fold change from vehicle-treated cells (error bars indicate s.e.m., *N*=5, *P<0.05 **P<0.01). (**f**) Chemo-tolerant cells generated from parent (DTC) and salinomycin-selected (Sal-DTC) MDA-MB-231 cells were analyzed by FACS for CD44^Hi^/CD24^Hi^, and results are expressed as % of total population (Data shown are mean±s.e.m., *n*=8, ANOVA analysis ***P<0.001, NS, not significant). (**g**) Graph shows mean fluorescent intensity (MFI) from FACS analysis of CD44 and CD24 expression in MDA-MB-231-parent, -DTC, -Sal-DTC or in a population of -expanded (E)-DTC and -Sal-DTC, demonstrating a reversal to parental phenotype when the chemotolerant cells are expanded over time (Data shown are mean±s.e.m. *n*=5, *P<0.05 **P<0.01). (**h**) Graph shows cell viability of each indicated population to docetaxel or doxorubicin, quantified by MTS cytotoxicity assay as % of viability in vehicle-treated control. All data shown are mean±s.e.m. from independent replicates (ANOVA analysis, *n*=8, ***P<0.001 versus parent cells).

To theoretically test the drug-induced phenotypic cell state transition versus clonal selection, we developed a phenotype switching model consisting of three cellular compartments, describing the population dynamics of CSCs (CD44^Hi^CD24^Lo^), the induced cells (CD44^Hi^CD24^Hi^) and non-stem cells (CD44^Lo^CD24^Hi^ and CD44^Lo^CD24^Lo^). Experimental data for the population dynamics were obtained from FACS data describing the re-equilibration kinetics of both the parental cells as well as the DTC, generated using the experimental design shown in Fig. 2a. The obtained parameter sets for the cases of the parental population and the DTC populations are summarized in Supplementary Table 2, using the methods described in detail in the Supplementary Information. In addition, Fig. 2b depicts the curves describing the time-evolution of the system composition from an arbitrary steady state, and highlights the system dynamics as it reaches equilibrium. In both cases, the model was able to fit well to the experimental data, implying that the model is versatile enough to describe the system dynamics of both treatment naive and post-chemotherapy cases, although, given the phenomenological nature of the model, we note that the derived parameter sets are useful only in a comparative sense, and are not necessarily precisely representative of the underlying biology for individual cases. Interestingly, the parameter values for either system was found to be quantitatively distinct, giving rise to different system saturations in equilibrium, that is, after induction of chemotherapy, there is a deterministic shift in the parameters governing the growth and switching rates of the subpopulations of cells, such that different steady states are observed. The model predicted that within the DTC population, the rates of proliferation of CSCs and induced CD44^Hi^CD24^Hi^ cells are significantly increased, whereas the rate of proliferation of non-stem cells decreases to a negligible value. The rate of transition from stem to non-stem cells remains the same in both environments, but the rate of transformation directly from non-stem to stem cells does not occur to a great degree in the chemoresistant cells. In addition, in the DTC, we observe high rates of transition between the induced CD44^Hi^CD24^Hi^ cells and non-stem cell compartments in both directions, indicating high inter-conversion (with no net direction), whereas in the case of the parental cells, the transition rate between the CD44^Hi^CD24^Hi^ cells and non-stem cells is highly skewed in the direction of the former, predominantly switching into the latter and not in the reverse direction. Finally, in the parental population, the CD44^Hi^CD24^Hi^ cells and CSCs are able to transition between each other. In contrast, in the DTCs, CSCs do not appear to transition into CD44^Hi^CD24^Hi^ cells, which are however able to transition into CSCs (Fig. 2c).

### Transient drug-tolerant phenotype originates from non-CSCs

To test the theoretical predictions that the CD44^Hi^CD24^Hi^ cells indeed arise from non-CSCs (that is, CD44^Lo^ cells), we sorted the parental population into four subsets based on various permutations and combinations of CD44^Hi^, CD44^Lo^, CD24^Hi^ and CD24^Lo^ status (Fig. 2a). These fractionated cellular subsets were then treated with high-concentration DTX for 48 h, following which the percentage of CD44^Hi^CD24^Hi^ cells in each population was quantified using FACS. In the absence of DTX, the cells re-equilibrate to a heterogenous cellular population similar to the treatment-naive parent cells with CD44^Hi^CD24^Hi^ cells forming ~20% of the total population, which was used as the baseline to elucidate the effect of DTX on each starting cellular fraction. Interestingly, as shown in Fig. 2d, a statistically significant increase in the CD44^Hi^CD24^Hi^ population was evident when the starting population consisted of CD44^Lo^CD24^Hi^ or CD44^Lo^CD24^Lo^ subsets.

We next used small molecule salinomycin-selection against CSC^23^ to significantly deplete the parent MDA-MB-231 population of CD44^Hi^CD24^Lo^ cells (Fig. 2e, Supplementary Fig. 4a). The parent and salinomycin-selected cells were then treated with DTX to generate DTCs, that were then FACS analyzed to quantify the percentage of CD44^Hi^CD24^Hi^ cells. The fact that both parent-derived DTCs and DTCs that were generated from salinomycin-selected cells (Sal-DTCs) exhibited similar percentages of CD44^Hi^CD24^Hi^ cells (Fig. 2f), despite the salinomycin-treated cells having ~50% less CD44^Hi^CD24^Lo^ cells to start with, indicated that the CD44^Hi^CD24^Hi^ were indeed originating from a non CD44^Hi^CD24^Lo^ population. Interestingly, the chemotherapy-induced upregulation of CD44 and CD24 levels were only transient, and in both DTCs and Sal-DTCs, the cells recalibrated back to parental CD44 and CD24 basal expression phenotype when expanded (DTC-E or Sal-DTC-E) over 35 days in the absence of chemotherapy pressure (Fig. 2g, Supplementary Fig. 4b shows schematic for FACS isolation). Although the cells existed in the transient CD44^Hi^CD24^Hi^ state, they were found to be resistant to high concentration of both DTX and doxorubicin. The expanded cultures, however, regained drug sensitivity (Fig. 2h), suggesting that the acquired tolerance to chemotherapy is reversible. Cell cycle analysis of the DTCs revealed that the cells were primarily in the G2M phase, with a large subset also undergoing endoreduplication, consistent with previous observations^24^. Interestingly, endoreduplication, the replication of DNA without undergoing an intervening mitotic division, has been reported to result in chemoresistance 25. Cell cycle analysis of the expanded DTCs revealed reversion to the parental phenotype, although a remnant tail of endoreduplicating cells was still evident (Supplementary Fig. 4c).

### Kinase library screening in DTCs

**Figure 3:**
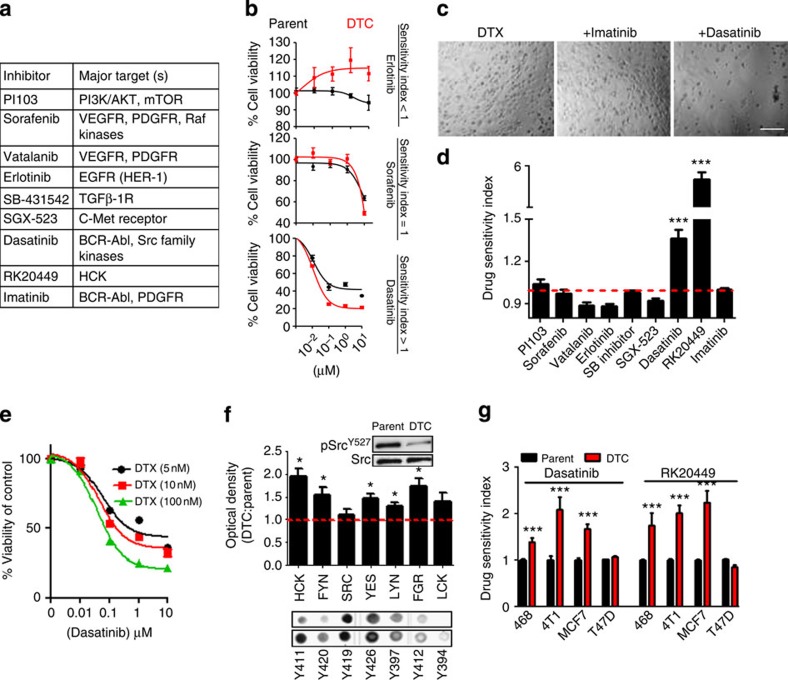
SFK/hck inhibitors preferentially disrupt a drug-tolerant state (**a**) A kinase inhibitor array was tested for activity against MDA-MB-231-DTCs. Table shows kinase inhibitors tested. (**b**) Representative concentration-effect curves showing activity of erlotinib, sorafenib and dasatinib on parent cells versus DTCs. Error bars indicate s.e.m. (**c**,**d**) Values obtained from concentration-effect analyses of cell viability were used to generate the sensitivity index (data shown are mean±s.e.m., *n*>25 independent experiments per group, ***P<0.001). Upper panel shows representative bright field microscopy of residual population. Scale bar, 100 μm (**e**) Viability curve following dasatinib treatment in MDA-MB-231-DTCs selected with increasing doses of docetaxel (5 nM, 10 nM 100 nM). (**f**) Phosphorylation arrays (lower panel) and quantification by optical density (graph) show fold change in the phosphorylation of residues corresponding to Src Family Kinases (SFK) in the MDA-MB-231-DTC versus parent cells. Inset western blot of the inactivating Src^Y527^ residue in DTCs versus parent cells (ANOVA analysis, *P<0.05, *n*=4). Full western blot images can be found in Supplementary files. (**g**) DTCs generated from a panel of basal-like and luminal breast cancer cells were treated with dasatinib or RK20449 (48 h), and drug sensitivity index (SI) was determined as described in methods. SI>1 indicates greater drug sensitivity compared with an equivalently treated parent population of cells (that is, greater sensitivity to the SFK inhibitors in the DTCs compared with parent).

To identify whether DTCs are therapeutically tractable during this transient phase, we performed a drug screen with a library of kinase-targeted agents (Fig. 3a). Although some targeted therapeutics, such as the Akt inhibitor, PI103, or the pan-kinase inhibitor, sorafenib, were non-selective for DTCs over parent cells (Sensitivity index~1), others like the EGFR inhibitor, erlotinib, inhibited parent cells, whereas the DTCs continued to grow (sensitivity index (SI) <1) (Fig. 3b). Interestingly, dasatinib, a dual SFK/BCR-Abl inhibitor exerted greater cell killing of DTCs compared with the parental fraction (SI >1) (Fig. 3b–d). In contrast to dasatinib, imatinib, a selective BCR-Abl inhibitor, had no effect on the DTCs, suggesting that the activity of dasatinib could be attributed to its SFK-inhibiting property (Fig. 3c,d). Interestingly, RK20449, a selective inhibitor of the SFK protein Hck^26^, was found to be ~600% more selective in reducing the viability of DTCs as compared with parental cells (Fig. 3d). Furthermore, dasatinib was found to exert a synergistic outcome against DTCs selected with increasing concentrations of DTX, suggesting that the refractory cancer cells exert a DTX dose-dependent reliance on the SFK-signalling pathway to persist during chemotherapy (Fig. 3e).

Consistent with the above results, a phosphorylation array revealed a global activation of the pro-oncogenic and pro-survival SFK family^27^ in the DTCs as compared with parent cells, with Hck as the predominant target (Fig. 3f). Western blotting revealed that the Src-activating residue (Y419) remained unchanged but the inactivating residue (Y527) was diminished in DTCs, indicating a gain-of-function mechanism underlying the activation of this pathway (Fig. 3f). Furthermore, Immunoprecipitation (IP) for phospho-Tyr Hck revealed a DTX concentration-dependent increase in phosphorylated Hck with maximal expression in the DTCs, which reverted back to parental levels by day 35 (in the expanded population) (Supplementary Fig. 5a). We next tested the efficacy of dasatinib and RK20449 against an array of basal and luminal parental breast cancer cell lines and the corresponding DTCs. As shown in Fig. 3g, the cell lines where treatment with DTX induced the CD44^Hi^CD24^Hi^ population were significantly more sensitive to SFK inhibition than the parent population. In contrast, this discrimination was lost in the luminal cancer cell line, T47D, which did not demonstrate an augmentation of the CD44^Hi^CD24^Hi^ population in the drug-tolerant subset. The addition of the BCR/ABL inhibitor, imatinib, with RK20449 did not further augment this sensitivity of DTCs to the latter, implicating only the SFK pathway in this response (Supplementary Fig. 5b). Furthermore, dasatinib treatment significantly inhibited the CD44^Hi^CD24^Hi^ population in the DTCs (Supplementary Fig. 5c). Taken together, these results suggested the involvement of the SFK pathway in mediating the transient chemotherapy tolerance arising owing to drug-induced phenotypic cell state transitions.

### CD44/CD24 clusters in lipid rafts with SFK/Hck

**Figure 4:**
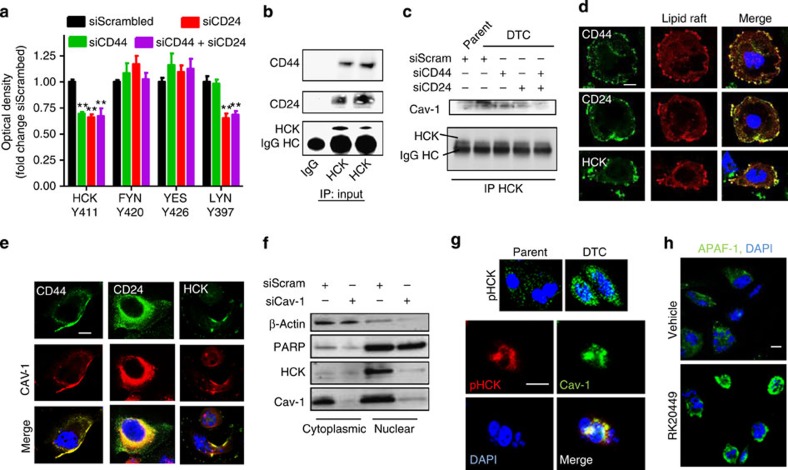
Mechanisms underlying SFK activation leading to adaptive resistance in DTCs. (**a**) Graph shows quantification of activated SFK in MDA-MB231 treated with high concentration docetaxel post siRNA knockdown of CD44, CD24 or both genes in (*N*=4, ANOVA **P<0.01). (**b**) Hck was immunoprecipitated from DTCs or parent MDA-MB-231s cell lysates followed by western blotting for CD44 and CD24 antibodies IgG input included for control. IgG HC indicates heavy chain (HC) bands. (**c**) Co-IP of Hck was performed from cell lysate of parent or DTCs generated from MDA-MB-231 cells transfected with siRNA targeting CD24 and CD44 or a combination of both. Western blotting indicates Cav-1 scaffolding to Hck. (**d**) Representative confocal images demonstrates colocalization of CD44, CD24 and Hck to lipid raft-rich regions of the cell membrane in DTC derived from MDA-MB-231 cells. Scale bar, 5 μm (**e**) Confocal microscopy identifies Caveolin 1 (Cav-1) colocalizing with CD24, CD44 and Hck in the MDA-MB-231 DTCs. Scale bar, 5 μm (**f**) Subcellular localization of Hck in DTC generated following siRNA-knockdown Cav-1. β-Actin and PARP indicate loading controls of cytoplasmic and nuclear compartments, respectively. (**g**) Confocal microscopy was used to identify subcellular localization in the nuclear plane of phosphorylated Hck (pHck) in the MDA-MB-231 parent compared with DTCs (upper panel). Dual staining shows a pattern of nuclear and perinuclear localized pHck and Cav-1 in the DTCs. Scale bar, 8 μm. (**h**) Representative confocal images reveal the expression of APAF-1 (Green signal) in MDA-MB-231 DTCs treated with vehicle or RK20449 (1 μM) for 24 h, and counterstained with DAPI. Scale bar, 8 μm.

As the DTCs exhibited increased activation of the SFK signalling, we used a short interfering RNA (siRNA)-based approach to test if the increased expressions of CD44 and CD24 are directly linked with the activation of SFK (knockdown validation can be found in Supplementary Fig. 5d). A phosphorylation array-based analysis of DTCs generated from cells treated with DTX following siRNA-knockdown of CD44 revealed a reduction in the phosphorylation of Hck. SiRNA-mediated knockdown of CD24 also decreased the phosphorylation of Hck and additionally of Lyn (Fig. 4a). Indeed, previous studies have implicated SFK proteins in mediating signalling through CD24 (refs 28, 29). Immunoprecipitation studies validated that both CD44 and CD24 scaffold with Hck (Fig. 4b). A double knockdown of CD44 and CD24, however, did not exert any additive effect, suggesting that the both contribute to the activation of SFK but do so through the same machinery (Fig. 4a).

To study the interactions between CD44, CD24 and SFK/Hck, we looked at the role of caveolins (Cav). Cav are major protein components of lipid rafts, and its upregulation is associated with poor prognosis in several human cancers^30^,^31^. Studies have implicated that engagement of CD44 and CD24 in lipid rafts can result in the activation of cortex kinases via clustering-mediated autophosphorylation^32^,^33^. Furthermore, recent studies have reported the activation of SFK by Cav-1 and vice versa^34^. Immunoprecipitation studies revealed an enhanced interaction between Hck and Cav-1 in the DTCs as compared with in the parents. Consistent with the earlier observation of the requirement of both CD44 and CD24 for the activation of Hck, we observed that siRNAs-mediated knockdown of either CD44 or CD24 is sufficient to inhibit this interaction (Fig. 4c). Interaction between Cav-1 and Hck was further evidenced by confocal microscopy (Supplementary Fig. 5b). Labelling the lipid rafts using a cholera toxin-based fluorescent tracer followed by confocal imaging revealed a robust colocalization of CD44, CD24 and Hck in the lipid rafts (Fig. 4d). Similarly, immunofluorescence imaging confirmed the colocalization of CD44, CD24 and Hck with Cav-1 in the DTCs. (Fig. 4e). Interestingly, the clustering of Hck with Cav-1 was found to facilitate a nuclear localization of the complex, which was augmented in the DTC compared with parent cells (Fig. 4f,g), and was blocked by a siRNA-mediated knockdown of Cav-1 (Fig. 4f). This is consistent with previous observations, where Cav-1 and SFK has been reported to facilitate stabilization and nuclear translocation of signalling proteins^35^. Nuclear translocation of activated Hck is reported to result in the inhibition of p73, resulting in a survival response via a reduced induction of the Caspase activation and recruitment domains 12/apoptotic protease-activating factor 1 (CARD-12/APAF1)^36^. Indeed, the inhibition of Hck with RK20449 released the suppression of proapoptotic CARD-12/APAF1 (Fig. 4h).

### Temporally sequenced SFK inhibitor and taxane *in vivo*

**Figure 5:**
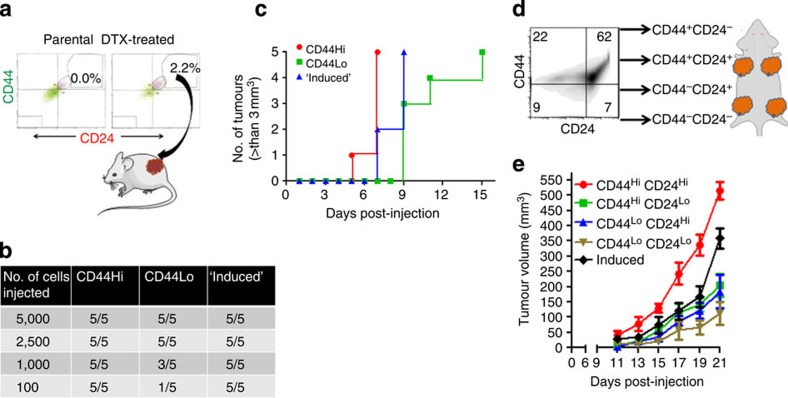
*In vivo* characterization of the tumorigenic property of chemotherapy-induced CD44^Hi^CD24^Hi^ cells. (**a**) Schematic shows the selection of ‘induced’ CD44^Hi^ CD24^Hi^ phenotype from 4T1 mammary carcinoma cells sorted by FACS. Cells that emerge *de novo* with a CD44^Hi^ CD24^Hi^ phenotype following chemotherapy treatment (50 nM docetaxel, 24 h) (~2.2% of the population that was not originally present in the parent population were considered an ‘induced’ subset. (**b**) Table shows the tumorigenicity of different subsets of breast cancer cells isolated on the basis of CD44, CD44 expression levels compared with the induced subset. Tumorigenicity was quantified by implanting different cell numbers in mice and monitoring the number of tumours developed. (**c**) Graph shows the temporal kinetics of tumour growth initiated from seeding CD44^Hi^, CD44^Lo^ or induced cells. (**d**) Schematic illustrates experimental design of *in vivo* kinetic analysis of tumour growth of distinct subpopulations. Cells were isolated from each quadrant and an equal number of cells were implanted in mice in separate areas. An induced CD44^Hi^CD24^Hi^ group was run in parallel. (**e**) Graph shows tumour volumes from indicated phenotypic subsets over time. Data shown are mean±s.e.m., *n*=4.

The *in vitro* results suggested a novel function of SFK signalling in breast cancer, driving a transient adaptive resistance during phenotypic cell state transitioning. We next investigated whether the inhibition of SFK could overcome adaptive resistance to taxanes *in vivo*. As the first step, we studied the tumorigenic ability of taxane-‘induced’ CD44^Hi^CD24^Hi^ cells compared with different phenotypic subpopulations of murine mammary carcinoma drug naive parental cells. The ‘induced’ cells were isolated using FACS based on the *de novo* appearance of a previously non-existent population (~2.2%) post acute cytotoxic pressure (Fig. 5a). We performed a dilution assay, where defined numbers of parental CD44^Hi^, CD44^Lo^ or the induced cells were implanted in mice. As shown in Fig. 5b, all the phenotypes could contribute to tumour progression in 100% of the animals when implanted in >2,500 cells. However, at the lower dilutions of 1,000 and 100 cells, only 60% and 20% of the CD44^Lo^ cells gave rise to tumours, respectively, consistent with previous observations that CD44^Lo^ cells are less tumorigenic. In contrast, both CD44^Hi^ and ‘induced’ CD44^Hi^CD24^Hi^ cells contributed to tumorigenesis in 100% of the animals even at the lowest dilution (100 cells). Monitoring the rate to tumorigenesis revealed that the parental CD44^Hi^ cells led to faster tumour development as compared with the induced cells, which in turn led to tumour development faster than the CD44^Lo^ cells (Fig. 5c). We next sorted the parental cells into CD44^Hi^CD24^Hi^, CD44^Hi^CD24^Lo^, CD44^Lo^CD24^Hi^ and CD44^Lo^CD24^Lo^, which were subsequently implanted in mouse at a dilution of 5,000 cells each. ‘Induced’ CD44^Hi^CD24^Hi^ cells were also injected at the same dilution (Fig. 5d). As shown in Fig. 5e, tumours derived from CD44^Hi^CD24^Hi^ cells were most aggressive in terms of tumour growth followed by the ‘induced’ CD44^Hi^CD24^Hi^ cells, whereas the tumours derived from CD44^Lo^CD24^Lo^ cells were the slowest growing. These results implicated that phenotypically transitioned chemotherapy-refractory cells can potentially reinitiate tumour growth.

**Figure 6:**
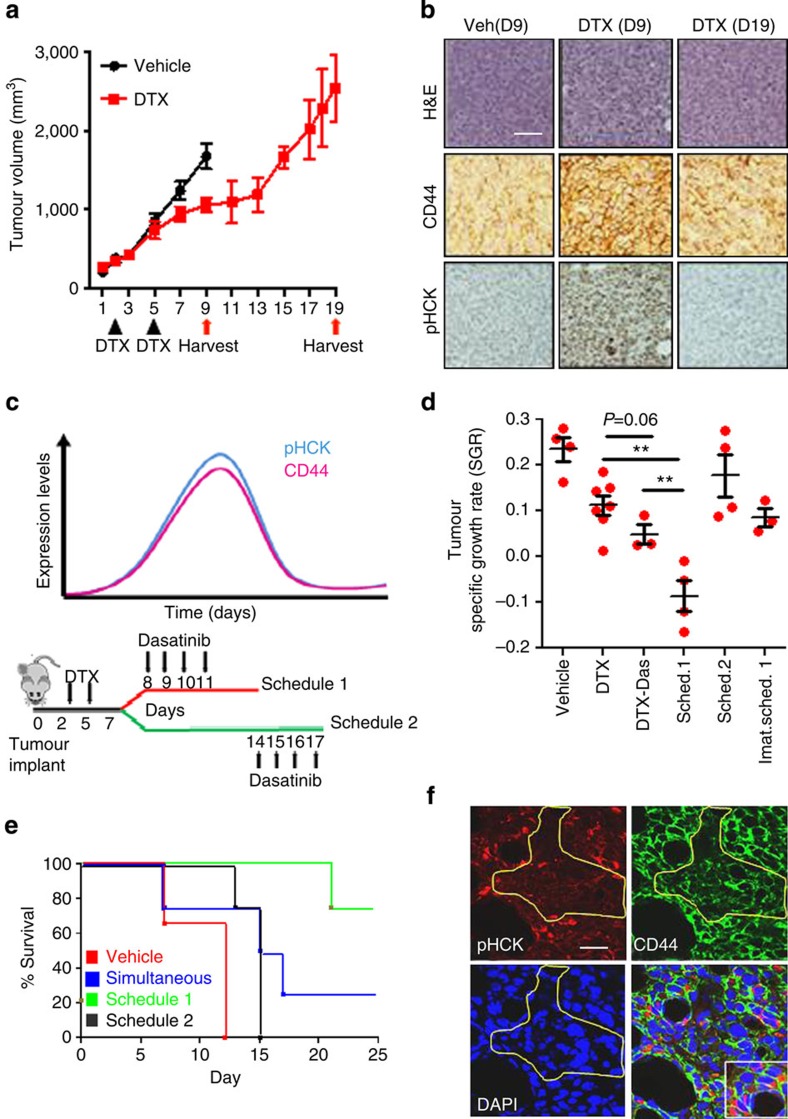
Sequential temporally constrained delivery of a taxane and SFK inhibitor regresses tumour growth rate and overcomes adaptive resistance. (**a**) Graph indicates tumour volume over time from heterotopic, syngeneic murine mammary carcinoma model (4T1). Groups were treated with docetaxel (DTX) or vehicle (black arrows show treatment days). Tumours were extracted on day 9 and 19 (Red arrows) corresponding to plateau or regrowth of tumour volume in docetaxel-treated arms, respectively (Data shown are mean±s.e.m., *n*=4). (**b**) Representative IHC of CD44 and pHCK following tumour extraction at indicated time points, H&E from serial sections confirm viable regions of tumour. Scale bar, 50 μm (**c**) Schematic shows experimental design for temporal delivery of dasatinib (10 mg kg^−1^) administered in two schedules, (1) 72 h or (2) 216 h post DTX treatment. The first schedule is designed to target the induction phase of chemotherapy-phenotypic transitioning and the second schedule targets the recalibration phase to parental state. (**d**) Histogram quantifies specific tumour growth rate. (**e**) Kaplan–Meier survival graph of an orthotopic syngeneic mammary carcinoma model treated as indicated (*N*=4 in all groups, *N*=3 for vehicle). (**f**) Representative confocal microscopy shows co-staining of CD44 and pHck. Regions of robust Hck activity correspond to regions of tumour with high expression of CD44 (areas outside yellow circumscription). Scale bar, 25 μm

We next studied whether the temporal induction of phenotypic cell state transition in response to chemotherapy can be recapitulated *in vivo*. Treatment-naive cells were implanted in a syngeneic 4T1 mammary carcinoma mouse model, which were then treated with a maximum-tolerated dose of docetaxel on days 2 and 5 post implantation. A control group was treated with the vehicle. As shown in Fig. 6a, a separation of the growth curves between treated and untreated group was evident by day 6, reaching a cutoff point by day 9 in the control group. The vehicle-treated animals and a batch of DTX-treated animals (during growth-plateau phase) were killed on day 9. The remaining drug-treated animals were subsequently killed on day 19, when the tumour growth rate (the slope of the curve) had reached the slope of growth observed in the vehicle-treated control group. Immunohistological and western blot analysis of the tumour tissue revealed a significant upregulation of CD44, phosphorylated Hck, as well as activated Src (via ablation of the inactivating residue at Y527) in the day 9 tumours from animals treated with DTX as compared with vehicle-treated controls. Furthermore, day 19 drug-treated tumours showed a reversal to the baseline (Fig. 6b, Supplementary Fig. 6a–d). This was consistent with the earlier *in vitro* observations, where chemotherapy induced a phenotypic switch towards a CD44^Hi^ state, with an associated activation of Hck, resulting in adaptive resistance (that is, generation of DTCs), and the transiently acquired phenotype recalibrating to the parental state with time.

Based on this understanding of the temporal induction of the phenotypic transition *in vivo*, we next explored whether a time-constrained administration of a SFK inhibitor could potentially reverse the adaptive drug-resistant state. The experimental design is outlined in Fig. 6c. The animals were treated with vehicle or DTX (at maximum-tolerated dose) on days 2 and 5 post implantation of tumour cells. The DTX-treated animals were then randomized into four groups. The first group was treated with four, once daily, doses of dasatinib, simultaneously administered with DTX between days 2 and 5. The second group was treated with dasatinib administered between days 8 and 11, that is, schedule 1, timed to target the induction phase of DTX-induced cell state transition. The third group was similarly treated with imatinib. The fourth group was administered with dasatinib between days 14 and 17 (Schedule 2), timed to target SFK during the reversion phase to parental phenotype. As shown in Fig. 6d (and Supplementary Fig. 6d), the simultaneous administration of DTX and dasatinib only marginally improved the antitumour efficacy compared with DTX alone treatment, whereas treatment with dasatinib as per schedule 2 had no statistically significant effect. Interestingly, dasatinib administered as per schedule 1 synergized with DTX in tumour growth inhibition. Imatinib, which was included as a negative control, had no effect on DTX-induced tumour growth inhibition. The Kaplan–Meier curves demonstrate that orthotopic tumour-bearing mice treated as per schedule 1 exhibited significantly superior survival than schedule 2, simultaneous administration and vehicle-treated controls (Fig. 6e). Consistent with the *in vitro* results, study of cross-sections of DTX-treated tumours revealed the localization of phosphorylated HCK with CD44^Hi^ cells. Furthermore, cells that had lower CD44 expression also exhibited low phosphorylated Hck (Fig. 6f). Finally, as shown in Supplementary Fig. 6e, treatment of animals with the Hck inhibitor administered post treatment with DTX treatment resulted in increased APAF1 expression that overlapped with TdT-mediated dUTP nick end labelling positivity, validating the role of Hck in suppression of apoptosis *in vivo*.

**Figure 7:**
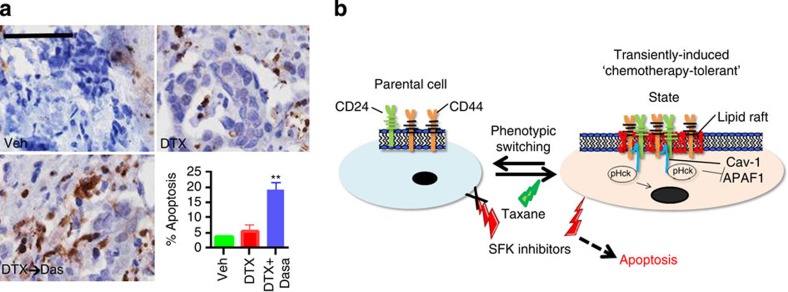
Temporally constrained inhibition of SFK signalling can target a vulnerable state during chemotherapy-induced cellular transition. (**a**) Representative H&E and IHC of activated caspase-3 in human taxane-refractory tumour explant treated with docetaxel or a combination of docetaxel and dasatinib. Histogram shows IHC score quantification of activated caspase-3 (*N*=6, **P<0.01). Data shown are mean±s.e.m. from independent replicates. Scale bar, 100 μm (**b**) Schematic shows the treatment of parent drug-naive cells with cytotoxic chemotherapy (taxane) confers phenotypic plasticity transitioning the population towards a transient drug-tolerant state, arising through clustering of CD44 and CD24 in lipid rafts, HCK activation and suppression of proapoptotic signalling via nuclear translocation. This transient state is vulnerable to inhibition of SFK using kinase inhibitors, leading to apoptosis. The same SFK inhibitors have no effect on parent cells.

At last, to study the clinical implications of these findings, we generated explants from primary tumour biopsies that were clinically resistant to DTX. The explants were treated with vehicle, DTX or a schedule of DTX followed by dasatinib. As shown in Fig. 7a, treatment with DTX did not cause a significant change in the percentage of apoptotic cells as compared with vehicle. In contrast, the sequenced dosing resulted in a marked increase in apoptosis. Taken together, these results suggest that targeting SFK/Hck during the chemotherapy-induced phenotypic cell state transition can overcome adaptive resistance.

## Discussion

In this study, we demonstrate that the introduction of temporality in the application of a chemotherapy drug pair can induce novel biological behaviour and an outcome that otherwise is not unmasked, if the two drugs are administered simultaneously or in the incorrect temporal window. We show that the treatment of breast cancer cells with SFK inhibitors immediately following a taxane-based chemotherapy results in an enhanced anticancer outcome. The first drug induces a phenotypic cell state transition from a non-CSC to a preferred CD44^Hi^CD24^Hi^ state, which can activate SFK signalling and confer adaptive resistance to chemotherapy. Interestingly, it is during this drug-induced phenotypic transition that the cells are also exquisitely sensitive to inhibition by a SFK inhibitor such as dasatinib. However, this sensitivity is lost when the cells recalibrate to the parental phenotype following removal of cytotoxic-chemotherapy pressure. This is consistent with recent clinical findings, where single-agent dasatinib did not exhibit significant antitumour activity in patients with heavily pre-treated metastatic breast cancer^37^,^38^, whereas dasatinib administered after cessation of DTX was found to be effective^39^.

Previous studies had reported CD44^Hi^CD24^Lo/−^ breast cancer cells as chemotolerant stem-like (CSCs) or basal-like state, whereas CD24^Hi^ cells were considered luminal and differentiated^40^. Interestingly, plasticity was reported between CD44^Hi^CD24^−^ cells and non-CSCs or CSC-depleted fractions, and the former could be enriched by chemotherapy^10^. Here we observed an increase in the CD44^Hi^CD24^Hi^ fraction following treatment with DTX. The increase in the expression of CD44 and CD24 in the explant cultures that were generated from a broad range of breast tumour types, and not just the basal type, indicated a chemotherapy-induced expression of CD44^Hi^ and CD24^Hi^ cells rather than an enriching of the CSCs. A similar increase was observed in both basal and luminal cell lines in response to DTX treatment *in vitro*, although it should be noted that the effects were more pronounced in the basal type cell lines. The use of CSC-depleted or CD44^Lo^ cells, using non-lethal chemotherapy concentrations, and mathematical modelling validated that this increase in the CD44^Hi^CD24^Hi^ fraction is indeed a result of *de novo* induction rather than selection of chemoresistant cells, and that these cells could arise from a non-CSC population. Interestingly, a membrane circumference CD24 expression, as observed in these ‘induced’ cells, is implicated in tumour progression and poor prognosis^41^. Furthermore, previous studies had shown that whereas CD44^Hi^ status is associated with increased risk of distant metastasis, the distant metastases that are frequently detected following chemotherapy are enriched for CD24^Hi^ cells^19^. Such observations could be similar to the phenotypic plasticity observed in this study. Indeed, recent studies have implicated stressor-induced phenotypic plasticity in driving the emergence of metastable phenotypic variants^42^.

Mechanistic studies revealed that the induction of CD44 and CD24 in the drug-tolerant CD44^Hi^CD24^Hi^ cells was associated with a colocalization in the lipid rafts, leading to an activation of the SFK signalling. CD44 engagement is reported to induce lipid raft coalescence to facilitate a CD44-Src-integrin-signalling axis, leading to increased matrix-derived survival^33^. Similarly, CD24 is known to augment c-src kinase activity and increase the formation of focal adhesion complexes in intact lipid rafts^32^. Of note, the Y419 residue was not differentially enhanced as expected based on previous reports^43^. We speculate this may arise as a consequence of phosphatase activity versus kinase activities within the DTC. Interestingly, in this study, the colocalization of both CD44 and CD24 was found to be critical for complexing with Cav-1, and the subsequent activation and subsequent perinuclear localization of SFK/Hck. Cav-1 is known to be overexpressed in aggressive breast carcinomas, and is also correlated with multi-drug resistance^44^. It is possible that increased apoptosis seen with the SFK and Hck inhibitors is a consequence of blocking Hck-mediated inhibition of p73 function^45^ and APAF-1 activation^36^. Interestingly, we did observe that a regimen containing carboplatin could induce apoptosis in the drug-refractory explants unlike DTX or doxorubicin, which could be a result of the ability of platinum-based cytotoxics to upregulate p73 (ref. 46). Platinum-containing regimens are being studied in chemotherapy-refractory triple-negative breast cancer^47^, and could potentially be useful in overcoming adaptive resistance to taxanes. Figure 7b summarizes the mechanisms underlying chemotherapy-induced phenotypic plasticity-driven adaptive response of the cells.

This study has several translational implications. First, it sheds newer insights into chemotherapy-induced adaptive resistance in breast cancer, where a taxane induces phenotypic cell state transition in the cells towards a transient CD44^Hi^CD24^Hi^ state. The clustering of CD44 and CD24 in the lipid rafts, and the complex with Cav-1 leads to the activation of SFK/Hck, which can confer adaptive resistance. It is possible that such transient drug-refractory states can confer an advantage for a fraction of cells to survive the initial onslaught of chemotherapy in the absence of stable resistance driven by Darwinian principles. Second, the understanding of the new role of SFK/Hck in mediating this phenotype also indicates that existing clinically approved drugs could potentially be repositioned for overcoming taxanes-induced adaptive resistance. Results from our explant studies, which closely mimic the clinical context, indicate that dasatinib, for example, can resensitize refractory breast cancer to taxane. Although the emphasis of this study has been on breast cancer, early data from other cancer cell lines indicate that this phenomenon could be ubiquitous. Finally, an interesting point to note is that the above signalling interactions are only triggered owing to drug-induced phenotypic cell state transition, and SFK inhibition has no effect in the absence of this phenotypic transition. The possibility of using a drug pair, administered in the correct temporal sequence, where the leading drug transitions a cancer cell to a phenotypic state vulnerable to the second agent opens up a new paradigm in the treatment of cancer.

## Methods

### Reagents

Unless noted otherwise, all reagents and drugs were of the highest grade purchased from Sigma-Aldrich (St Louis, MO, USA). Vincristine was purchased from Tocris biosciences (Minneapolis, MN, USA). Cabazitaxel, PI103, Dasatinib, Doxorubicin, LY294002 and Erlotinib were purchased from LC Labs (Woburn, MA, USA).

### Cell culture and gene knockdown with siRNA

MCF-7 (American Type Culture Collection; ATCC), MDA-MB-231 (ATCC), SKBR3 (ATCC) and SUM159 (Asterand, Detroit, MI, USA) were cultured in DMEM containing 10% fetal bovine serum, MDA-MB-468 (ATCC), T47D (ATCC) and 4T-1 mammary carcinoma cells (ATCC) were cultured in RPMI containing 10% fetal bovine serum (Invitrogen, Carlsbad CA, USA) at 37 °C and 5%CO_2_. In total, 4306 and 4412 ovarian cancer cell lines were a kind gift from Dr Daniela Dinulescu, BWH. During treatments with chemotherapeutics, cells were grown to semi-confluence and treated with indicated concentrations of chemotherapy in serum-containing medium for indicated time points. For siRNA gene knockdown, cells were plated at a concentration of 5 × 10^4^ cells ml^−1^. Pre-validated Silencer Select siRNA targeting (sense sequences) pan-CD44(1) (5′- UAUUCCACGUGGAGAAAAAtt -3′) panCD44(2) (5′- GCGCAGAUCGAUUUGAAUAtt -3′) panCD24 (5′- GGAGAGGAACAUCCAAAAtt -3′) Cav-1 (5′- GCUUCCUGAUUGAGAUUCAtt -3′) were purchased from Ambion (Invitrogen, Grand Island, NY, USA), and were transfected using lipofectamine 2000 (Invitrogen) following manufacturer’s protocol. Scrambled siRNA was used as a control.

### Cell culture and generating DTC

Cancer cells were plated at a density of 0.5–1 × 10^5^ cells ml^−1^ and allowed to adhere for 24–48 h. When cells reached ~70% confluency, they were treated with cytotoxic drugs at indicated concentrations for 4–48 h and utilized for subsequent assays. Following washes with PBS, adherent cells were trypsinized and re-plated at a density of 1.5–2 × 10^5^cells ml^−1^ and cultured in serum-containing medium. After 24 h incubation, floating cells were removed and remaining cells were washed with 1 × PBS and considered as chemotherapy-tolerant cells. Expander populations were cultured in fresh media replaced at routine intervals over a 35-day period.

### Cytotoxicity and cell viability assays and calculation of drug sensitivity index

Parent cells, DTC or an expanded population of DTC were generated as described and plated at a concentration of 1.5 × 10^5^ in a clear bottom 96-well plate. Cells were exposed to treatments for 48 h in serum-containing medium. Following incubation, cells were washed with PBS and recovered in serum and phenol red-free RPMI or DMEM and subsequently treated with MTS reagent using manufacturer’s protocol (Promega, Madison, WI, USA). Drug SI was derived as follows: cell viability was determined as % of vehicle control for treatment conditions of 10 nM, 100 nM 1,000 nM and 10 μM of indicated drug, and the values were averaged across these four drug concentrations. SI was calculated as a ratio of this average value for parent:DTC. SI=1 correlates to parental sensitivity, SI<1 correlates to resistance compared with parent cell line and SI>1 correlates to enhanced sensitivity to the indicated drug compared with the parent cell line at the same concentration average. Validation of cytotoxicity was performed by bright field microscopy and trypan blue co-stain.

### Human explant studies

Human breast cancer biopsy tissues (*N*=14) from anonymous patients with varying stages of disease, receptor status and prior treatment history (Supplementary Fig. 1 shows patient history) were obtained from HCG Bangalore Institute of Oncology, Kidwai Memorial Institute of Oncology, Mazumdar Shaw Cancer Center under institutional review board approval. The tumour samples were transported to the laboratory at 4 °C, in appropriate transfer buffer for *ex vivo* studies and molecular and pathological evaluation. Tissues were cut into thin sections and cultured in 96-well plates that were coated with tumour matrix proteins and media supplemented with 2% autologous serum. Tumours were treated with taxanes, doxorubicin or platinum-containing regimen for 72 h at concentrations based on reported clinical Cmax values^20^. For this study, the concentrations used were 3.4 μM DTX and 5.6 μM doxorubicin. After treatment, tumour cell viability was measured by cleaved caspase-3 determined by immunohistochemistry (IHC) score. CD44 (clone IM7) and CD24 (clone ML5) were used at a dilution of 1:50.

### *In vivo* experiments

All *in vivo* experiments were performed in compliance with Institutional Animal Care and Use Committee protocol approved by Harvard Medical School and in accordance with institutional guidelines, supervised on-site by veterinary staff. 4T-1 mouse mammary carcinoma cells (10^6^ cells) suspended in 100 μl PBS were injected into the flanks of 5–6-week-old female Balb/C mice (heterotopic) or mammary fat pads (orthotopic) (Charles River, Wilmington, MA, USA). DTX was dissolved in pure ethanol at a concentration of 50 mg ml^-1^ mixed 1:1 with Polysorbate 80 (Tween 80) and brought to a final working concentration with 5% glucose in PBS. Once tumours became palpable (~100 mm^3^), DTX or vehicle treatments were administered at 100 μl volumes. Dasatinib was dissolved in DMSO to working concentration and delivered as 50 μl injections on indicated days. RK20449 was dissolved directly in PBS and administered i.p. at 30 mg kg^−1^, twice daily for 3 consecutive days. Tumour volumes were measured by a third party unaware of treatment conditions using digital calipers (Starlett, Athol, MA, USA), and tumour volumes were calculated by the following formula: (width × width × length)/2 and expressed as mm^3^ or relative volume increase from day 1. Tumour-specific growth rate^48^ was calculated by the algorithm (ln[*V*_2_/*V*_1_]/[*t*_2_−*t*_1_]) where *V*=volume and *t*=time in days. At the end of study, tumour cell lysis was done by homogenization of equal weight tissue sections incubated in 3 × RIPA buffer containing 2 × protease/phosphatase inhibitor cocktail (Thermo Fisher, Waltham, MA, USA). CD44 western blotting was performed with a mouse-specific antibody (clone ABIN135065, Antibodies Online, Atlanta GA, USA, 1:500 dilution) conjugated to Biotin (Thermo Fischer). All *in vivo* experiments were performed in compliance with Institutional Animal Care and Use Committee protocol approved by Harvard Medical School.

### Phosphorylation arrays

The Proteome Profiler (R&D systems, Minneapolis MN, USA) was used to identify phosphorylated residues correlating to SFK-associated proteins. Following the Bradford protein analysis assay to normalize total protein content, cell lysate was applied to the phosphorylation membranes following manufacturer’s protocol. Western blot of total protein (Akt and Src) was used to confirm equal loading of lysate. Membranes were visualized by chemiluminescence (Syngene, Cambridge, UK). Optical densities were determined by Image J software (NIH.gov) and Adobe CS5. Reference spots were used to normalize between array membranes.

### FACS analyses and cell cycle

Cells were cultured as indicated and fixed with 4% paraformaldehyde in PBS for 30 min at room temperature (RT) and blocked in 10% goat serum (v/v), 0.05% saponin was used to permeabilize cells when necessary. Following PBS washes, cells were incubated with CD24-PE and CD44-APC (BD biosciences, San Jose, CA, USA) for 60 min at RT or overnight at 4 °C and analyzed by FACS (Accuri cyomteters Inc. Ann Arbor, MI, USA). Single-stain controls were used to set gating parameters and any compensations. AnnexinV/PI was analyzed following manufacturer’s protocol (BD biosciences). All FACS results were analyzed by FlowJo software following a rigorous doublet discrimination based on FSC:A versus width as well as FSC:A versus height (Tree Star Inc., Ashland, OR, USA). Analyses were also performed through Accuri cFlow plus software to obtain and confirm mean fluorescent intensity (GNU.org). Cell cycle analysis was performed as follows: cells were generated as described, following two washes with 1 × PBS, cells were trypsinized and collected. Permeabilization was achieved by incubation in 70% ethanol overnight at 4 °C. Cells were then washed two times with 1 × PBS and incubated with RNAase A for 15 min at 37C followed by propidium iodide solution for 30 min at 4C (Genscript USA Inc. Piscataway, NJ, USA). Cells were read at excitation/emission 594/535 by Flow cytometry (BD Accuri C6, BD biosciences). All results were analyzed by FlowJo flow cytometric analysis and cell cycle analysis software following a rigorous doublet discrimination based on FSC:A versus width as well as FSC:A versus height. Cell sorting was performed on live cells. In brief, cells were incubated with fluorescent antibody for 20 min at RT in PBS. Following washes, cells were sorted by FACS (BD FACS Aria IIU Special Order, BD biosciences). Schematic in Supplementary Fig. 4a shows example of sorting ‘induced population of DTC’ and schematics in Fig. 5 show example of sorting a chemotherapy-induced subset for *in vivo* analyses (defined as a population of cancer cells, which harbour a CD44+/CD24+ phenotype not originally present in the parent populations).

### Immunohistochemistry

Tumour tissues were fixed in Phos stop (Roche, Basel, Switzerland) containing 4% buffered formalin and embedded in paraffin. Before immunohistochemical staining of target proteins, 4-μm-thick tissue sections mounted in poly L-lysine-coated glass slides were deparaffinized and rehydrated. Heat-induced antigen retrieval was achieved using citrate buffer (pH7.8). The sections were soaked in Antigen Unmasking Solution (Vector Laboratories, Burlingame, CA, USA) for 10 min followed by retrieval using a microwave for 25 min. Endogenous hydrogen peroxidase was blocked by incubating the sections with 3% H_2_O_2_ (Merck) for 15 min and washed in running tap water for 3 min followed by a wash in 1 × TBS for 7 min. After initial blocking of the slides in 10% normal goat serum (Vector Laboratories) for  h at RT, tissue sections were incubated with primary antibodies for additional 1 h at RT. Following primary antibodies were used: anti human Ki-67 (rabbit polyclonal from Vector Laboratory, 1:600 dilution), anti-human cleaved caspase3 (rabbit polyclonal, clone D175, Cell Signaling Technology, Cambridge, MA, USA), Anti-human CD44 (Clone IM7), P-Hck^Y410^ (Cell Signaling Technology). Secondary antibody (Signal Stain Boost IHC Detection Reagent, horseradish peroxidase, Rabbit, Cell Signaling Technology) was added to the sections and incubated for 45 min at RT and washed four times in 1 × PBS for 3 min each. Appropriate isotype-matched immunoglobulin G controls were included for each secondary antibody. Chromogenic development was done by exposure of tissues to 3,3′-diaminobenzidine substrate (DAB Peroxidase Substrate Kit; Vector Laboratories). Images of immunostained sections were visualized by Leica DM4000 microscope at × 200 or × 400 magnifications and images were acquired. Immunoreactivity was scored by intensity of staining (0, no staining; 1, weak; 2, moderate; 3, strong) and percentage of positive cells. By multiplying both values, a final score was calculated. Scoring was performed in a blinded fashion by two experienced pathologists. IHC performed from frozen sections were fixed with 10% formalin and permeabilized with 0.05% saponin or fixed and permeabilized with ice-cold methanol. Frozen section IHC was visualized by confocal microscopy as described below. Immunohistochemical images shown in figures chosen as representative are derived as examples determined by an experienced pathologist in each case to reflect overall alterations in tissue staining/architecture of the respective experiments performed. Tumours from at least four individual mice per group were used for IHC, and were evaluated from at least 25 individual fields per group at shown magnifications. Quantification of data for selected IHC can be found in supplementary information. For human explant IHC, representative images were obtained from 14 individual patients

### Confocal and immunofluorescence microscopy

Cells were generated as described above and plated in four chamber glass slides (BD Biosciences) at a concentration of 100,000 cells ml^−1^. Following treatments, cells were washed in PBS and fixed in 4% paraformaldehyde for 30 min. Permeabilization, when necessary, was achieved with 10% (v/v) goat serum (Vector Laboratories) and 0.05% Saponin (w/v) in PBS for 90 min. Blocking was performed in 10% (v/v) goat serum in PBS. The cells were labelled with the indicated primary antibodies CD44 (Clone IM7 from eBioScience) conjugated to FITC (AnaSpec, Freemont, CA, USA) at 1:100, CD24 (BD biosciences) conjugated to Fluor 594 (Anaspec), unconjugated antibodies were incubated and followed by a secondary antibody conjugated with Alexa Fluor 488 or Alexa Fluor 594 (Invitrogen) at 1:250 and masked with DAPI-containing hard-set mounting medium (Vector Laboratories). Bright field and fluorescent images were obtained using three channels on a NIKON Eclipse TI-U microscope with a × 20 ELDW, × 10 or × 40 Plan-Apo objective lens (Nikon, Melville, NY, USA). NIS Elements Viewer version 3.22 (Nikon) software was used to capture the images to file. Confocal microscopy was performed with an inverted Nikon Confocal microscope (TE2000) with Auto DeVlur deconvolution software and fitted with three laser detection (Nikon). Gains were set manually based on negative control stains (secondary antibody only) and were left unaltered between treatment groups of similar experiments. Visualization of lipid rafts by confocal microscopy was achieved using Vybrant Lipid raft staining kit (fluor594) obtained from Life Technologies according to manufacturer’s protocol. TdT-mediated dUTP nick end labelling staining was performed to visualize regions of apoptosis using the TUNEL assay kit and performed as indicated by provider (Roche). When representative images are shown in figures, these are derived from experiments performed in at least biological triplicate on independent occasions. In general, images were obtained from >100 cells per conditions and chosen to represent the overall alterations in each experimental group.

### Protein expression and interaction studies

Laemmli sample buffer was prepared as a 5 × solution containing β-mercaptoethanol as a reducing agent. Immunoprecipitaion was performed using both classic and direct IP kits purchased from Pierce following manufacturer’s protocols (Thermo Fisher inc. Rockford, IL, USA). In brief, cell lysates were prepared using IP/Lysis buffer (Thermo Fisher inc.) in the presence of 2 × HALT protease/phosphatase inhibitor cocktail (Thermo Fisher inc.). For classic immunoprecipitation, lysates were combined with indicated antibodies for 48 h at 4 °C and combined with protein A/G agarose beads for 4 h before elution with 2 × Laemli buffer at 100C. Direct immunoprecipitation was performed following manufacturer’s protocol. In brief, antibodies were covalently attached to agarose beads, lysate was combined with antibody-agarose bead conjugates for 24 h before washes and elution with provided Elution buffer. Protein samples were resolved by SDS–PAGE and transferred to polyvinylidene difluoride membranes before incubation at 4 °C with indicated primary antibodies; Hck, p-Tyr, Src pY527, PARP, Hck, cleaved caspase-3 and β-Actin were purchased from cell signaling technology. Cav-1 was purchased from BD biosciences. Polyvinylidene difluoride membranes with primary antibody were incubated at RT with horseradish peroxidase-conjugated secondary antibodies (BD Ann Arbor) and resolved by chemiluminescence using the G-Box and Syngene software (Syngene). When possible, blots were stripped (Thermo Fischer, Rockford IL) and re-probed with a second primary antibody. Optical densities of western blots were measured using ImageJ open source software (National Institutes of Health) and validated using Adobe CS5. Nuclear and cytoplasmic isolation was performed using the subcellular fraction kit following manufacturer’s protocol (Thermo Fisher inc.). Total PARP antibody (Cell signaling) or β-Actin were used to control loading from nuclear and cytoplasmic compartments, respectively. Western blotting images chosen as representative depictions in the figures demonstrate equivalent results taken from biological replicates (N>3). Full blot images in the main and supplemental figures, which were cropped for figure preparation, can be found in a separate supplemental figure. Molecular weight ladders have been inserted graphically as shown.

### Mathematical modelling

To theoretically test the drug-induced phenotypic plasticity versus clonal selection, we developed a phenotype switching model consisting of three cellular compartments, describing the population dynamics of CSCs (*S*) (CD44^Hi^CD24^Lo^), the induced (*I*) cells (CD44^Hi^CD24^Hi^), and non-stem (*NS*) cells (CD44^Lo^CD24^Hi^ and CD44^Lo^CD24^Lo^). The model consists of nine parameters, three of which describe the net proliferation rates for each of the compartments, and the remaining six parameters describe the transition rates between the compartments (that is, the rates of cells switching from one cellular subtype to another). We denote the number of CSCs, non-stem cells and induced CD44^Hi^CD24^Hi^ cells at the time *t* by *S*(*t*), *N*(*t*), *I*(*t*), respectively. We take *ρ*_*k*_ to be the (net) reproductive rate of cell compartment *k*. We let *ρ*_*ij*_ be the rate of transfer of cells from compartment *i* to compartment *j*.


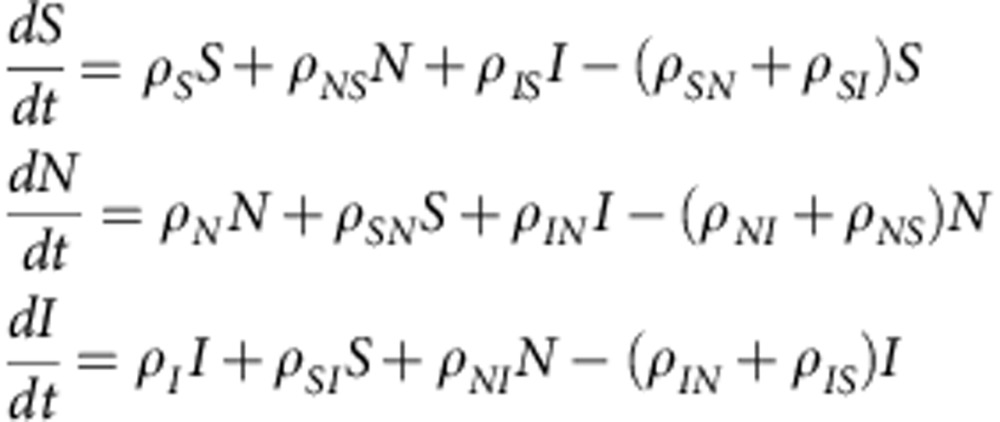


By using FACS data (described above), the experimental data obtained were summarized into time-dependent curves describing the proportion of each cellular subpopulation over time. These data were then combined with the model, for both the parental cell experiments as well as the ‘mimic’ experiments (independently). The model parameters were then fitted to the experimental data using the PotterWheel toolbox for Matlab, which uses numerical methods to fit parameter sets, describing data most accurately. It was noted that the final day experimental values for the parental cells (at FACS day 3), and for the CD44^Hi^CD24^Hi^ (at FACS day 1) produced values representative of the steady state cellular proportions for each of the systems, so while fitting for parental cells gave a best fit value with such a steady state immediately, fitting with the CD44^HI^CD24^HI^ values did not. Therefore, when fitting to the experimental data, the steady state was restricted to values near the day 1 values, by adding a data point to be fitted with the same values as reported on day 1, but at large time. The results obtained using this additional data point gives the reported values for the CD44^HI^CD24^HI^ parameters, providing a realistic experimental steady state. The bounds set on each of the parameter values, when fitting, were restricted between 0 and 0.5 for the net proliferation rates, because experimentally it was observed that there was a great deal of cell death, thereby necessitating reduced net proliferation rates. The bounds for the transition rates were restricted to be between 0 and 1, because these rates represent the proportion of cells undergoing a transition at any given time, it was felt that there was no experimental observation, supporting the further restriction of these parameters.

### Statistics

Statistical analysis was carried out with Prism software (Graphpad, LaJolla, CA, USA). Experimental data is expressed as mean±s.e.m., and analyzed using analysis of variance followed by Bonferroni post test or Student’s *t*-test.

## 

## Author contributions

A.G. and S.S. designed the study, analyzed the data and wrote the manuscript. B.M. and P.K.M. conducted human explant experiments. A.G. and S.R. performed *in vitro* and *in vivo* studies. D.G. performed pathology analysis. A.D. and M.K. developed the mathematical model.

## Additional information

**How to cite this article**: Goldman, A. *et al*. Temporally sequenced anticancer drugs overcome adaptive resistance by targeting a vulnerable chemotherapy-induced phenotypic transition. *Nat. Commun.* 6:6139 doi: 10.1038/ncomms7139 (2015).

## Supplementary Material

Supplementary Information Supplementary Figures 1-7 and Supplementary Tables 1-2,
