# Supplementary material for: Temporally sequenced anticancer drugs overcome adaptive resistance by targeting a vulnerable chemotherapy-induced phenotypic transition
Source: Nat Commun. 2015 Feb 11;6:6139. doi: 10.1038/ncomms7139 (PMC4339891; doi:10.1038/ncomms7139)
Supplement: Supplementary Information — Supplementary Figures 1-7 and Supplementary Tables 1-2, [file ncomms7139-s1.pdf]

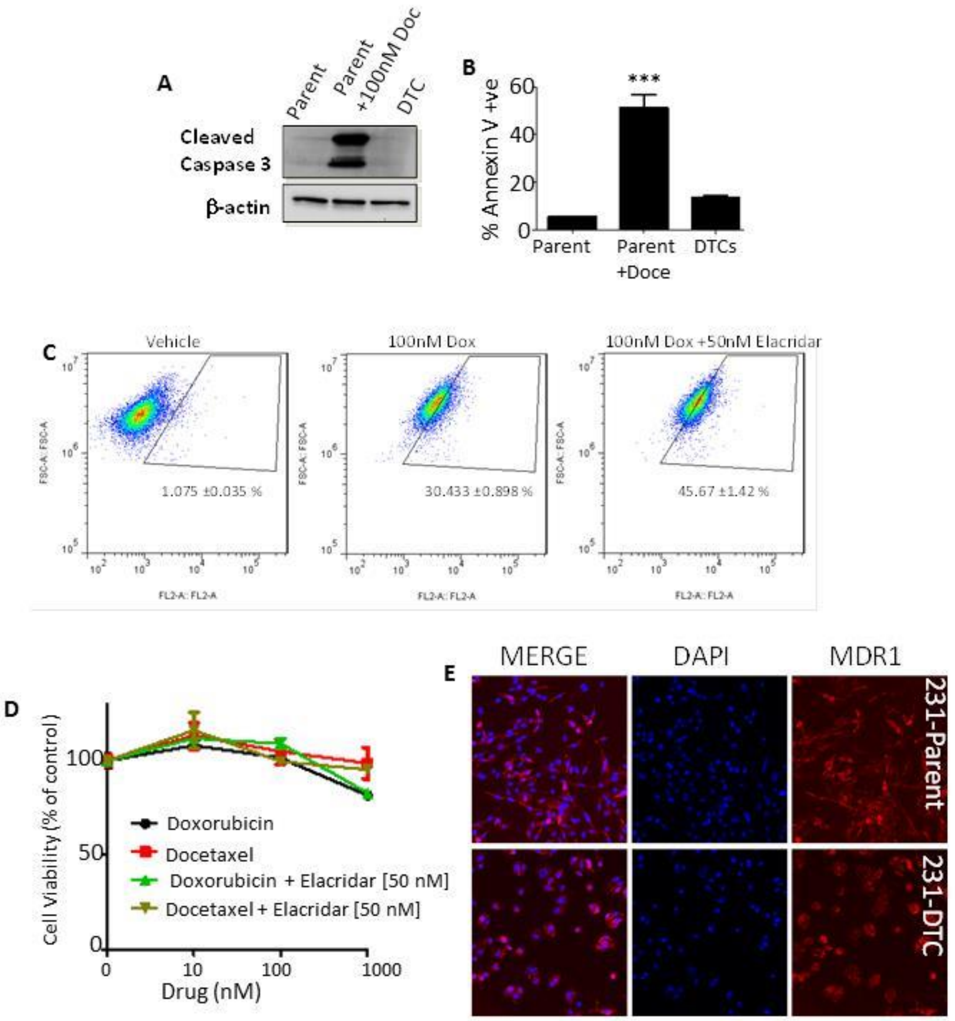

Supplemental Fig 1. DTCs exhibit adaptive resistance against both taxanes and anthracyclines, which is not mediated via a pgp-transport. A) A representative Western blot analysis shows increased cleaved caspase-3 levels in docetaxel-treated MDA-MB-468 parent cells (100nM 48hr) vs in untreated parent cells or in DTCs derived from the MDA-MB-468 cells following high dose chemotherapy. Full western can be found in Suppl. Fig. 7 **B)** Histogram shows quantification of apoptotic cells in each population as determined by FACS analysis of AnnexinV/PI (mean fluorescence of apoptosis vs. untreated parental control). **C)** FACS analysis of doxorubicin (100nM) internalization in the presence or absence of 50nM Elacridar evaluated at 24h. **D)** Cell viability analysis of DTC incubated in the presence of indicated chemotherapy ± the PgP transport inhibitor, elacridar [50nM]. Data shown are mean SEM from a minimum of three independent replicates ***P<0.001 using ANOVA). **E)** Representative epifluorescence images of MDA-MB-231 parent or DTCs showing the expression of the PgP-1 protein (MDR-1).

A

Tumor Origin	Cell line	Classification	Mean Fluorescence of CD44 in DTC subset (% increase of parent)
Breast	MDA-MB-231	Triple negative; Metastatic and invasive	214 ± 46
Breast	MDA-MB-468	Triple negative; Metastatic	179 ± 37
Breast	MDA-MB-435	Triple negative; Metastatic and invasive	208 ± 147
Murine Mammary Carinoma	4T-1	Triple neg.; Metastatic and invasive	177 ± 33
Ovarian	4306	ER, PR positive K-ras$^{G12D/+}$/Pten$^{-/-}$; Metastatic and invasive	268 ± 10
Ovarian	4412	K-ras$^{G12D/+}$/Pten$^{-/-}$; Metastatic and invasive	270 ± 7.0

B

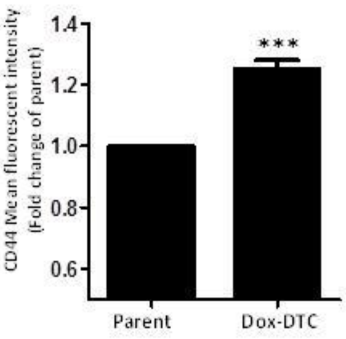

Supplemental Fig 2. Treatment with cytotoxic chemotherapy induces CD44 expression in DTCs generated using a range of cell lines. A) Table shows increase in expression of CD44 following treatment of different cell lines with docetaxel. The DTCs were analyzed by FACS for level of CD44 expression quantified by normalized mean fluorescent intensity (MFI) and expressed as % fold change from parent cells. **B)** Graph shows increase in CD44 expression in DTCs generated following treatment of MDA-MB-231 cells with doxorubicin (Dox). Data shown are mean ± SEM (***P<0.001 vs parent, t-test).

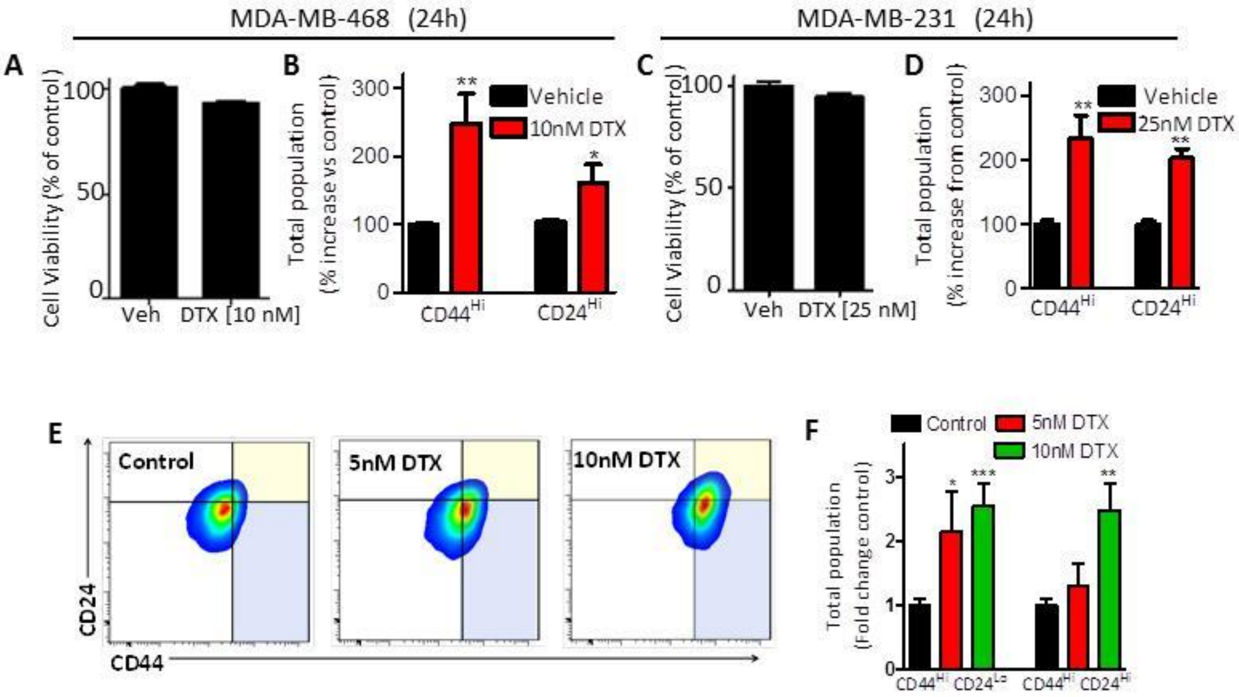

Supplemental Fig. 3. Docetaxel concentration-dependent induction of CD44 and CD24 in breast cancer cell lines. A) Graph shows cell viability of MDA-MB-468 TNBC metastatic cells treated *in vitro* with docetaxel (DTX) at 10nM for 24h. **B)** Graph shows increase in CD44 and CD24 expressions in MDA-MB-468 cells treated with docetaxel (DTX) at 10nM (24h). This design of acute chemotherapy treatment was used throughout the study as a method of evaluating chemotherapy-induced alterations without subjecting cells to a chemotherapy-selection process. Similar experiments were performed on MDA-MB-231 cells using a higher dose of chemotherapy (25nM). Graphs shows effect of docetaxel (DTX) at 25nM (24h) on **C)** cell viability and **D)** increase in CD44 and CD24 expressions in MDA-MB-231 TNBC metastatic cells. FACS analysis for expression of CD44 and CD24 determined in 20% highest [Biomarker(X)^{Hi}] population in parent (control) vs cells treated docetaxel (DTX), maintaining equivalent gating parameters between each group values, was used to calculate % change in the markers (N=6, *p<0.05 **p<0.01 compared to vehicle treated cells). **E)** Representative FACS analysis indicates the proportion of cells in $CD44^{Hi}/CD24^{Lo}$ (transparent blue quadrant) or $CD44^{Hi}/CD24^{Hi}$ (transparent yellow quadrant) with increasing concentration of chemotherapy in MDA-MB-468 cells (24h). **F)** Graph shows quantification of these values were determined from independent replicates and expressed as % of parent population, fold increase (histogram). Data shown are mean ± SEM (*p<0.05, **p<0.01, ***p<0.001).

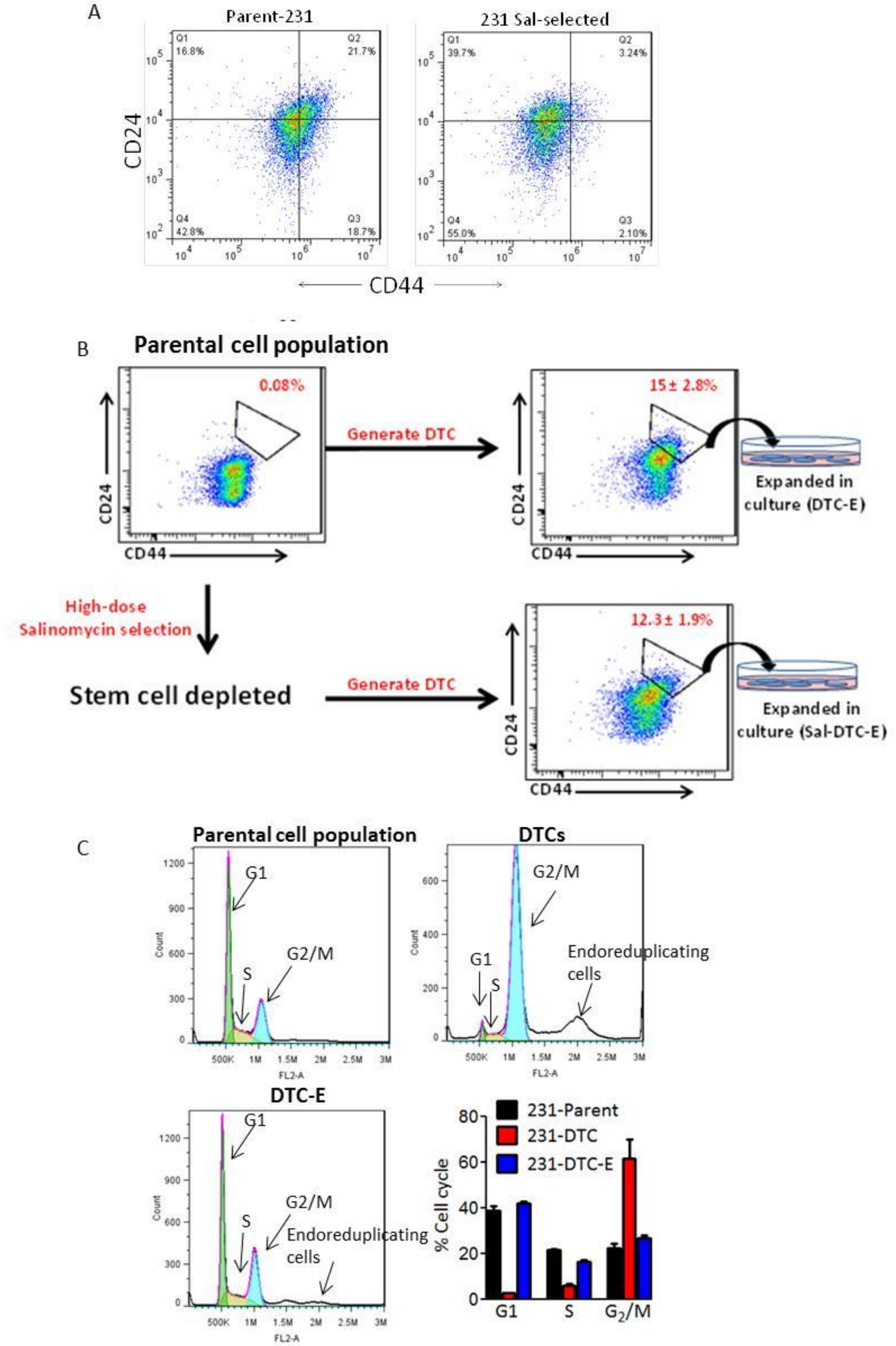

Supplemental Fig. 4. Schematic showing experimental design in generation of DTCs from parent (with intact CSC pool) or salinomycin-treated (CSC-depleted) parent pool. A) Representative FACS of salinomycin-treated MDA-MB-231 to illustrate depletion of the $CD44^{hi}CD24^{Lo}$ population. **B)** Representative FACS and descriptive schematic illustrates cell sorting $CD44^{Hi}/CD24^{Hi}$ cells from DTCs generated from docetaxel-treated parent cells or docetaxel-treated cells that were first depleted of CSCs by salinomycin (Sal-DTCs). A gate was set in the vehicle-treated parent cell lines characterized by an absence of a $CD44^{Hi}/CD24^{Hi}$ population (0.08%). Generation of DTC resulted in the shift of cells to constitute a new population which emerged within this gate of $CD44^{Hi}/CD24^{Hi}$. These cells were isolated by FACS sorting and further cultured in vitro to generate the expanded populations of DTC-E and Sal-DTC-E (populations were expanded for ~35-40 days and performed in biological replicates). (C) Cell cycle distribution of parental, DTCs and DTC-Expanded populations of MDA-MB-231cells. DTCs and DTC-Es were generated as described earlier using docetaxel treatment. Graph shows quantification of the percentages of cells in each stages of cell cycle. Data shown are mean ± SEM (n=3).

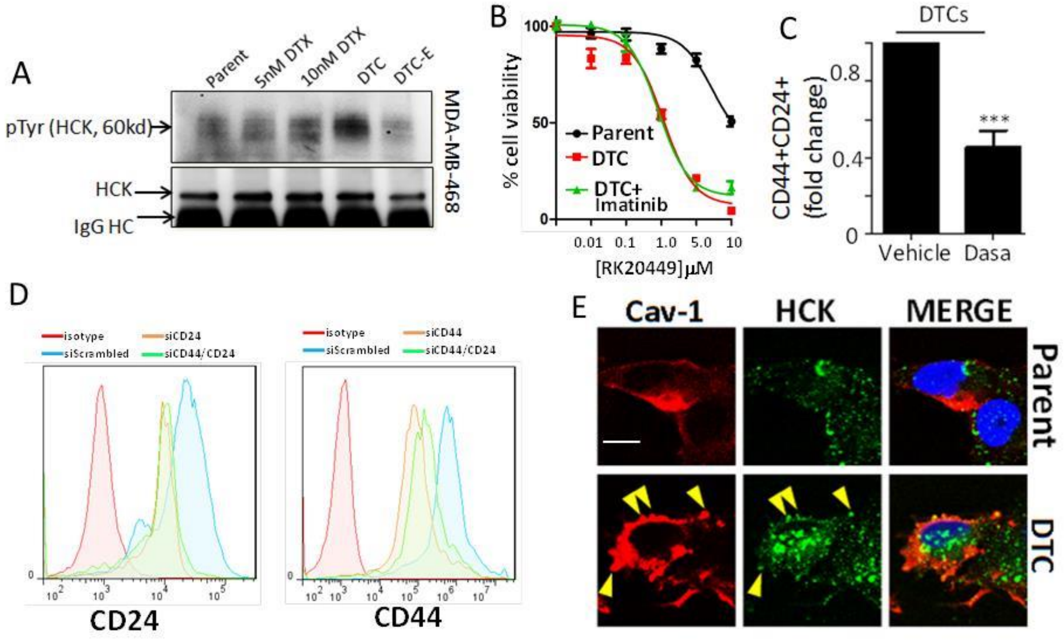

Supplemental Fig. 5 Enhanced Hck signaling in drug tolerant cells confers a survival advantage A) Docetaxel induces a concentration-dependent phosphorylation of Hck. A representative co-precipitation of HCK from MDA-MB-468 parent treated with 5nM or 10nM docetaxel (24h) or a population of DTCs (generated using 100nM docetaxel) and DTC-Es, followed by Western blot indicates phosphorylation of the HCK protein band. IgG heavy chain indicated at 55kd vs. Hck expressed at 60kd. DTCs show maximal pHck, which reverts back to parental state in the DTC-Es. Full western can be found in Suppl. Fig. 7 **B)** Graph shows cell viability of parent cells and DTCs following treatment with increasing concentrations of the Hck inhibitor, RK20449. Addition of Imatinib (10μM) to RK20449 treatment shows no additional benefit suggesting SFK-specificity with no bcr/Abl component (Data shown are mean ± SEM, n=10 independent replicates). **C**) MDA-MB-231-DTCs were treated with dasatinib (10μM) for 48hrs. Survivors were processed by FACS for $CD44^{Hi}/CD24^{Hi}$. Quantification of this population was measured between vehicle treated and dasatinib treated DTC (Data shown are mean ± SEM, ***p<0.001). **D)** Confirmation of gene knockdown using siRNA targeting CD44/CD24 or a dual knockdown indicated in MDA-MB-231. **E)** Representative confocal images shows co-localization of HCK and Cav-1 in MDA-MB-231-parent and -DTCs. Scale bar = 6μm

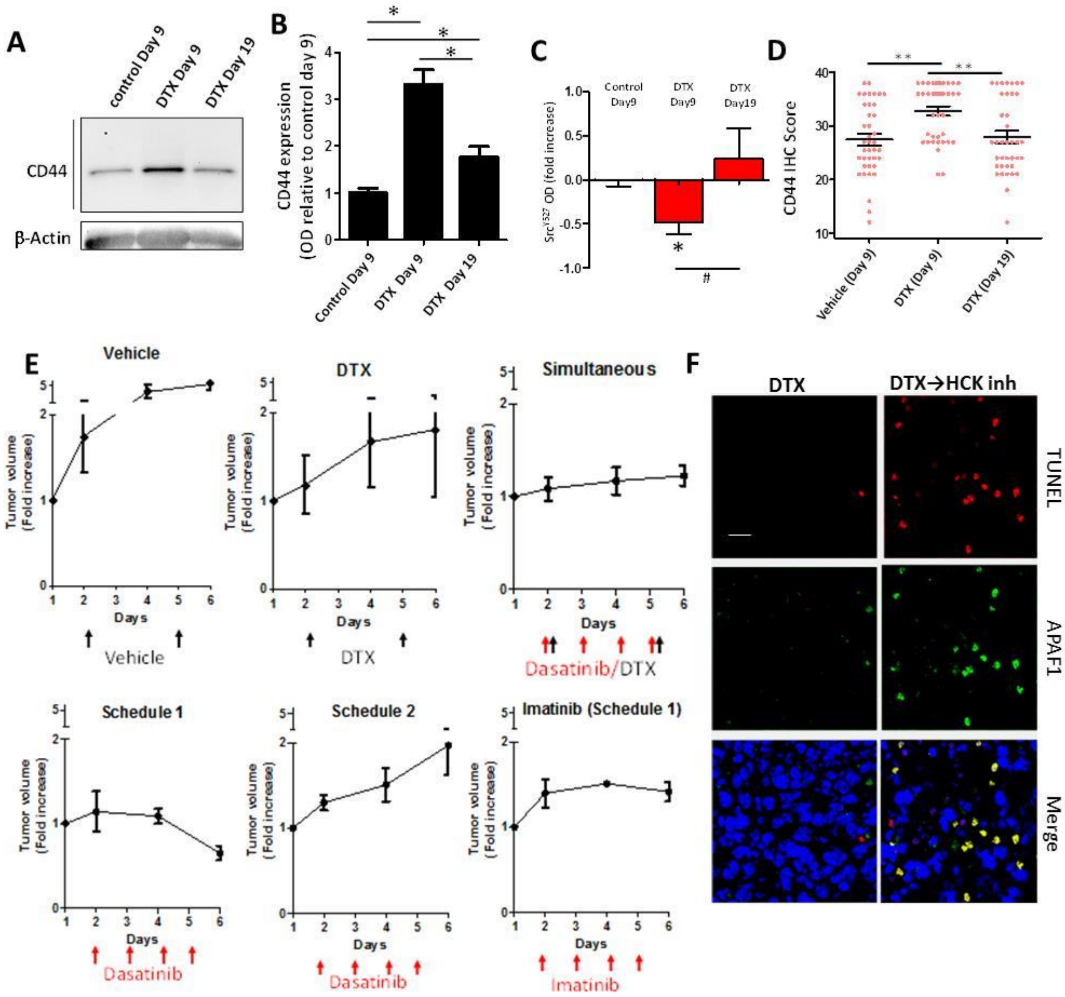

Supplemental Fig 6. Drug-induced phenotypic plasticity in vivo. A) Representative Western blot of CD44 from pooled tumor tissue homogenate from at least 3 mice of each control day 9, DTX-treated day 9 or 19. **B)** Optical density of CD44 protein expression was determined from Western blots performed on 3 separate tissue homogenates and expressed as fold change from control day 9. (*$p<0.05$ between indicated groups, error bars indicate SEM of biological replicates). **C)** Quantification of optical density of immunoblot from tumor extract processed for level of the inactivating residue of Src (Y527). Error bars indicate SEM from biological replicates (*$p<0.05$ compared to vehicle control, #$p<0.05$ between indicated groups). **D)** Quantification of CD44 expression correlating to main figure 3E. IHC score was determined by a pathologist in a blinded fashion as described in methods section. **E)** Tumor volumes for treatment groups of indicated regimens from mammary carcinoma heterotopic model (4T1). Relative volume changes were used to calculate specific growth rates shown in main figure 6. **F)** Representative epifluorescence images show greater apoptosis in the Hck inhibitor-treated animals. Mice bearing orthotopic syngeneic tumors (N=4) were treated with 2 doses of DTX (10mg/kg i.v.) or vehicle and subsequently treated with RK20449 (30mg/kg i.p. 2x/day for 3 days) or vehicle (PBS). Tumors were harvested on day 3 and evaluated for coincidence of TUNEL positivity and APAF-1. Scale bar = 25μm

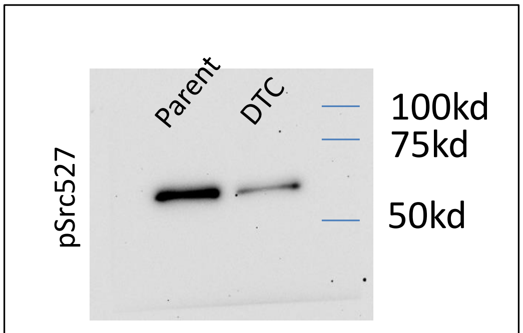

Main Figure 3F.

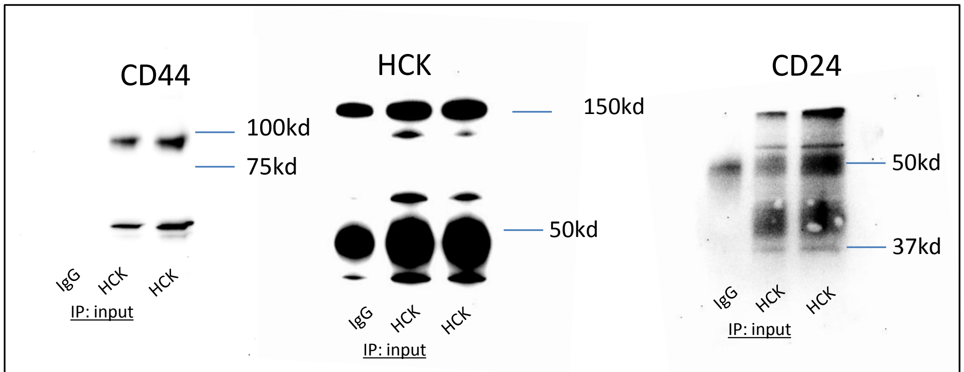

Main Figure 4B.

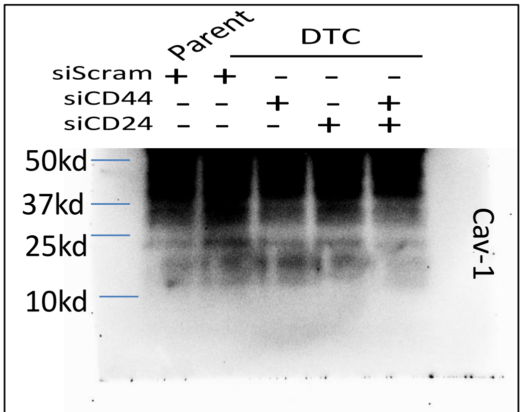

Main Figure 4C.

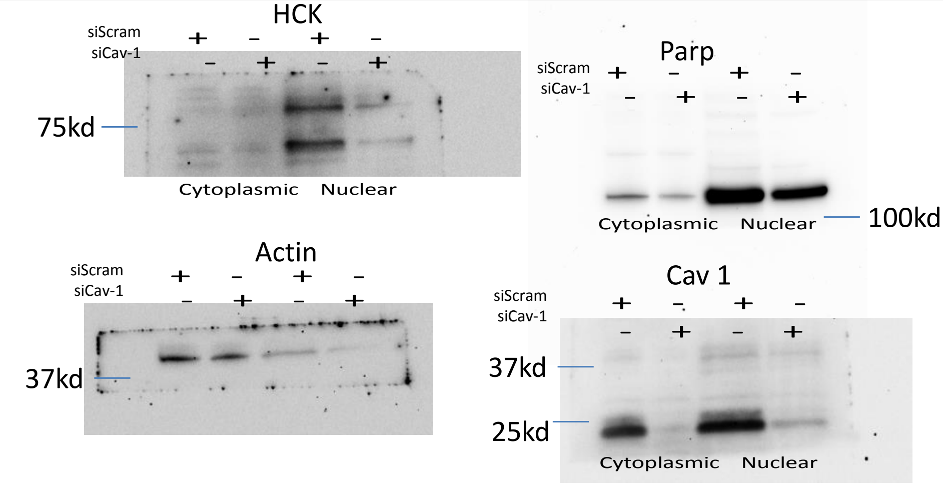

Main Figure 4F.

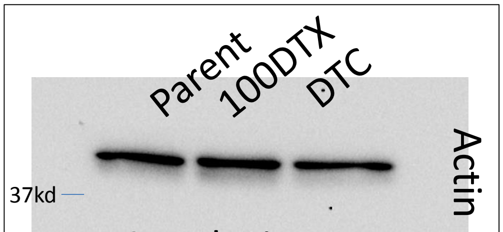

Suppl. Fig. 1

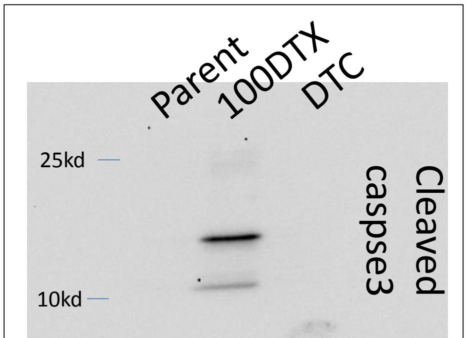

Suppl. Fig. 1

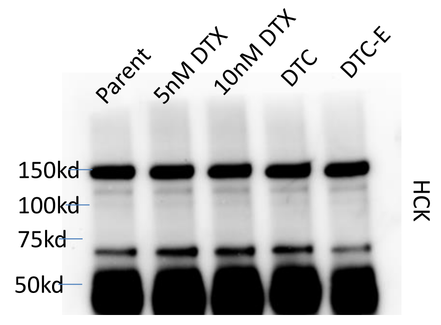

Suppl. Fig. 5A

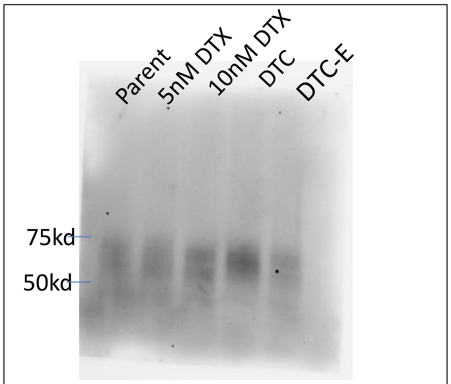

Suppl. Fig. 5A

Supplementary Figure. 7. Complete blots of images used in the manuscript.

Supplemental Table.1 Disease stage, pathological classification, and prior clinical history of the tumor biopsies used in explant studies.

Patient ID	Age/Sex	Tumor stage	Receptor Status	Treatment
Pt. 1	33/F	Stage2	$ER^{+}PR^{+}$ $HER2^{-}$	4 cycles of Adriamycin + Cyclophosphamide + 5-FU<br>3 cycles of Paclitaxel + Adriamycin+ Cyclophosphamide
Pt. 2	35/F	Stage3	ER^{+} PR^{+} $HER2^{-}$	6 cycles of Docetaxel + Epirubicin + Cyclophosphamide<br>2 cycles of Docetaxel + Zoledronic acid
Pt. 3	43/F	Stage4	ER-PR+ HER2-	4 cycles of cyclophosphamide + doxorubicin
Pt. 4	46/F	Stage4	ER+PR+HER2+	N/A
Pt. 5	45/F	Stage4	ER+PR+HER2-	6 cycles of Epirubicin + 5-FU + Cyclophosphamide
Pt. 6	38/F	Stage3	ER+PR-HER2-	3 cycles of Epirubicin + 5-FU + Cyclophosphamide
Pt. 7	28/F	N/A	ER+PR+HER+	N/A
Pt. 8	37/F	Stage4	ER-PR-HER-	6 cycles of Docetaxel + Epirubicin + Cyclophosphamide
Pt. 9	70/F	Stage2	ER-PR-HER2-	6 cycles of Epirubicin + 5-FU + Cyclophosphamide
Pt. 10	37/F	N/A	ER-PR-HER2-	6 cycles of Adriamycin+ Cyclophosphamide + Docetaxel
Pt. 11	55/F	N/A	ER-PR-HER-	6 cycles Adriamycin + Cyclophosphamide + Docetaxel
Pt. 12	51/F	Stage4	ER^{-} $PR^{-}HER2^{+}$	6 cycles of Paclitaxel + Adriamycin + Cyclophosphamide
Pt. 13	52/F	Stage3	ER-PR-HER2+	5-FU + Epirubicin + Cyclophosphamide
Pt. 14	35/F	N/A	ER-PR-HER2+	3 cycle of 5-FU + Epirubicin + Cyclophosphamide

Supplemental Table. 2 Growth and transition rate parameters as determined from in vitro experimental data under basal and chemotherapy pressure. A phenotype switching model consisting of three cellular compartments describing the population dynamics of CSCs (*S*) ($CD44^{Hi}CD24^{Lo}$), the induced (*I*) cells ($CD44^{Hi}CD24^{Hi}$), and non-stem (*NS*) cells ($CD44^{Lo}CD24^{Hi}$ and $CD44^{Lo}CD24^{Lo}$) was developed. The model consists of nine parameters, three of which describe the net proliferation rates for each of the compartments, and the remaining six parameters describe the transition rates between the compartments (i.e. the rates of cells switching from one cellular subtype to another). The number of CSCs, non-stem cells, and induced $CD44^{Hi}CD24^{Hi}$ cells at the time t are denoted by $S(t),\ N(t),\ I(t)$, respectively. ρ_k is the (net) reproductive rate of cell compartment , ρ_{ij} is the rate of transfer of cells from compartment i to compartment j.

Parameters	Normal Parameter Value (Parental population data, no chemotherapy)	Parameter value when measuring transition from normal to 24h after application of chemotherapy
ρ_S	0	0.5
ρ_N	0.5	0
ρ_I	0	0.29
ρ_{SN}	1	0.96
ρ_{NS}	0.55	0
ρ_{NI}	0.21	1
ρ_{IN}	1	0.98
ρ_{SI}	0.58	0
ρ_{IS}	0.96	0.38

Parameter	Description
$S(t)$	Number of cancer stem cells at time t
$N(t)$	Number of non-stem cells at time t
$I(t)$	Number of induced $CD44^{Hi}CD24^{Hi}$ cells at time t
ρ_S	Rate of symmetric stem cell division
ρ_N	Rate of symmetric non-stem cell division
ρ_I	Rate of symmetric induced cell division
ρ_{SN}	Rate of transition from stem to non-stem cells
ρ_{NS}	Rate of dedifferentiation from non-stem to stem cells
ρ_{NI}	Rate of transition from non-stem to induced cells
ρ_{IN}	Rate of transition from induced to non-stem cells
ρ_{SI}	Rate of transition from stem to induced cells
ρ_{IS}	Rate of transition from induced to stem cells
